# Addressing biases and limitations in feature attribution for circRNA modification profiling

**DOI:** 10.1093/bib/bbag168

**Published:** 2026-04-08

**Authors:** Souichi Oka, Kota Takemura, Yoshiyasu Takefuji

**Affiliations:** Research and Development Planning Department, Science Park Corporation, 3-24-9 Iriya-Nishi, Zama-shi, Kanagawa 252-0029, Japan; Research and Development Planning Department, Science Park Corporation, 3-24-9 Iriya-Nishi, Zama-shi, Kanagawa 252-0029, Japan; Department of Data Science, Faculty of Data Science, Musashino University, 3-3-3 Ariake Koto-ku, Tokyo 135-8181, Japan

**Keywords:** circularRNA, epitranscriptomics, machine learning, feature importance, model bias

## Abstract

Li *et al*. (CircRM: Profiling circular RNA modifications from nanopore direct RNA sequencing. *Brief Bioinform* 2026;**27**:bbaf726.) introduced Circular RNA Modifications (CircRM), a computational framework employing eXtreme Gradient Boosting and SHapley Additive exPlanations (SHAP) to profile RNA modifications in circular RNAs, achieving high predictive accuracy. However, we argue that strong predictive performance does not validate the biological reliability of the resulting feature-importance rankings. In heterogeneous feature spaces, tree-based models exhibit inherent biases, favoring continuous, high-cardinality variables—such as genomic position—over sparse sequence patterns, potentially obscuring true biological determinants. Furthermore, reliance on SHAP introduces theoretical vulnerabilities; recent findings on attribution limitations indicate that baseline sensitivity can decouple explanations from local mechanistic behavior. To address these analytical pitfalls, we advocate for a robust framework incorporating Highly Variable Gene Selection and Feature Agglomeration to mitigate multicollinearity, complemented by model-agnostic non-parametric methods such as Spearman’s rho and Kendall’s tau. Adopting these strategies ensures that computational profiling yields biologically actionable insights rather than reflecting statistical artifacts.

Dear Editors,

Li *et al*. introduced CircRM, a computational framework that combines eXtreme Gradient Boosting (XGBoost) with SHapley Additive exPlanations (SHAP) to profile RNA modifications in circular RNAs (circRNAs) from raw nanopore direct RNA sequencing data [1]. Using this framework, they reported 427 high-confidence circRNAs—substantially exceeding commonly used baseline approaches—and developed modification-detection models with Receiver Operating Characteristic (ROC) Area Under the Curve (AUC) values of 0.855 (m5C), 0.817 (m6A), and 0.769 (m1A). A SHAP-based analysis further highlighted several highly ranked predictors, such as relative position within the annotated 5' Untranslated Region (UTR) and distance to nearby DRACH motifs, which were used to motivate reported differences between linear-RNA and circRNA modification patterns. Collectively, these results suggest that CircRM is a promising tool for characterizing the circRNA epitranscriptome at single-molecule resolution; however, the validity and biological interpretability of the resulting feature-importance rankings warrant further discussion.

This concern follows well-recognized distortions in end-to-end analytical pipelines that can decouple predictive performance from mechanistic biological interpretation. Reliance on predictive accuracy as a proxy for biological feature relevance has been repeatedly cautioned against, particularly in high-dimensional transcriptomic settings where correlated predictors enable models to exploit statistical regularities that need not reflect causal determinants [2, 3]. The risk is amplified when training labels are defined through probabilistic score thresholds, in which case the model may preferentially learn threshold-induced proxies rather than bona fide mechanistic signals. As summarized in our Supplementary Material, a survey of more than 300 peer-reviewed studies reports systematic skew in feature-importance estimates under closely related conditions. Accordingly, strong AUC values alone provide limited assurance that the ranking of predictors reflects distinct biological mechanisms.

Tree-based learning algorithms, including XGBoost, also exhibit method-specific biases in feature-importance estimation [4–6]. In particular, split-based learning can favor variables that facilitate early partitioning, an effect that can be exacerbated by multicollinearity and complex dependency structure in transcriptomic feature spaces [7]. In Li *et al*.’s specific modeling setting, several highly ranked predictors—such as relative positions within annotated UTRs/Coding Sequence (CDS) and distances to nearby motifs—may be susceptible to this issue, thereby complicating their mechanistic interpretation. Moreover, CircRM employs a heterogeneous feature space that mixes dense continuous nanopore-signal statistics (e.g. mean, dwell time) with sparse one-hot encoded sequence features. In such mixed feature spaces, importance can be preferentially attributed to continuous, high-cardinality variables, potentially obscuring subtle but biologically decisive sequence patterns. Given the absence of definitive ground truth for circRNA-specific feature relevance in RNA modifications, and the difficulty of disentangling correlated sequence effects, the reported rankings should be interpreted with appropriate caution.

In addition to correlation-driven concerns, SHAP-based attribution can be vulnerable to theoretical limitations that constrain the reliability of post hoc explanations in certain settings [8, 9]. Recent results sometimes referred to as ‘impossibility’ theorems indicate that, under particular desiderata for additive attributions, local explanatory faithfulness can be fundamentally limited, depending on modeling assumptions and the choice of baseline or reference distribution [10]. In practice, the completeness constraint can induce attributions that reflect global properties of the fitted model and background distribution rather than strictly local behavior around a given observation. Consequently, SHAP values may be sensitive to baseline specification, and their magnitude or sign need not map straightforwardly onto mechanistic influence in the underlying biological system. In the CircRM application context, features identified as key predictors (e.g. distance to DRACH motifs) could therefore partially reflect baseline- or distribution-sensitive effects rather than direct modification determinants. This possibility is particularly salient where explanations are used to motivate read-level mechanistic narratives, and it motivates explicit validation beyond attribution scores alone.

To address these analytical pitfalls, a more robust inferential framework is required—one that does not hinge on model-dependent attributions and that explicitly addresses the structural complexity and redundancy of sequencing-derived features. We therefore propose combining Highly Variable Gene Selection with Feature Agglomeration to mitigate multicollinearity and feature redundancy [11, 12]. In particular, for high-dimensional sequence-derived inputs such as the one-hot encoded 5-mer representations used in CircRM, these procedures can consolidate correlated patterns and reduce the arbitrariness with which importance is distributed across redundant nucleotide indicators. In addition, complementing these steps with non-parametric association measures—specifically Spearman’s rho and Kendall’s tau—enables the assessment of monotonic and rank-based relationships without assuming linearity, thereby providing a more interpretable, model-agnostic perspective on feature–signal associations [13, 14].

In summary, while CircRM exhibits strong predictive performance, the biological interpretation of its feature-importance rankings remains provisional. The combination of algorithm-specific biases in tree-based models and the context-dependent limitations of SHAP indicates that highly ranked predictors may, in part, reflect statistical structure rather than causal mechanisms. Predictive accuracy should therefore not be conflated with explanatory adequacy. Progress in circRNA epitranscriptomic profiling will benefit from validation strategies that emphasize robustness, stability, and model-agnostic corroboration to ensure that computational inferences translate into biologically actionable insight.

**Key Points**

- Although CircRM demonstrates certain levels of predictive performance, such metrics may not necessarily ensure the reliability of the resulting feature-importance rankings for RNA modifications.
- Tree-based models like XGBoost are prone to specific biases in high-dimensional settings, which can prioritize high-cardinality features over biologically decisive sequence patterns.
- The application of additive attribution methods, such as SHAP, warrants careful consideration as rankings can be sensitive to baseline specifications rather than directly reflecting mechanistic biological influence.
- To mitigate these issues, we advocate for a robust analytical framework primarily based on Highly Variable Gene Selection and Feature Agglomeration, complemented by non-parametric statistical methods to provide a model-agnostic perspective.

## Supplementary Material

Supplementary_material_bbag168

## Data Availability

No new data were generated or analyzed in support of this research.
