# Supplementary material for: Addressing biases and limitations in feature attribution for circRNA modification profiling
Source: Brief Bioinform. 2026 Apr 8;27(2):bbag168. doi: 10.1093/bib/bbag168 (PMC13069895; doi:10.1093/bib/bbag168)
Supplement: Supplementary_material_bbag168 [file supplementary_material_bbag168.docx]

**Supplementary Material**

Over 300 peer-reviewed articles from the National Library of Medicine:

Google Search Syntax (site:nih.gov):

"bias in feature importance" OR "bias in feature selection" OR "biases in feature importance"

OR "biases in feature selection" OR "biased feature importance" OR "biased feature

selection" OR "feature importance bias" OR "feature selection bias" site:nih.gov

1. Abdollahi, A., Li, D., Deng, J., & Amini, A. (2024). An explainable artificial-intelligence-aided safety factor prediction of road embankments. Eng. Appl. Artif. Intell., 136(Part A), 108854. https://doi.org/10.1016/j.engappai.2024.108854
2. Abdulrauf Sharifai, G., & Zainol, Z. (2020). Feature Selection for High-Dimensional and Imbalanced Biomedical Data Based on Robust Correlation Based Redundancy and Binary Grasshopper Optimization Algorithm. Genes, 11(7), 717. https://doi.org/10.3390/genes11070717
3. Adeyeye, O. A., Hassaan, A. M., Yonas, M. W., Yawe, A. S., Nwankwegu, A. S., Yang, G., Yao, X., Song, Z., Kong, Y., Bai, G., & Zhang, L. (2025). Integrating partial least square structural equation modelling and machine learning for causal exploration of environmental phenomena. Environ. Res., 274, 121358. Advance online publication. https://doi.org/10.1016/j.envres.2025.121358
4. Adler, A. I., & Painsky, A. (2022). Feature Importance in Gradient Boosting Trees with Cross-Validation Feature Selection. Entropy, 24(5), 687. https://doi.org/10.3390/e24050687
5. Aghaei, F., Tan, M., Hollingsworth, A. B., & Zheng, B. (2016). Applying a new quantitative global breast MRI feature analysis scheme to assess tumor response to chemotherapy. J. Magn. Reson. Imaging, 44(5), 1099–1106. https://doi.org/10.1002/jmri.25276
6. Akshay, A., Besic, M., Kuhn, A., Frauenfelder, T., Eberli, D., & Sulser, T. (2024). Machine Learning-Based Classification of Transcriptome Signatures of Non-Ulcerative Bladder Pain Syndrome. Int. J. Mol. Sci., 25(3), 1568. https://doi.org/10.3390/ijms25031568
7. Alaimo Di Loro, P., Scacciatelli, D., & Tagliaferri, G. (2023). 2-step Gradient Boosting approach to selectivity bias correction in tax audit: an application to the VAT gap in Italy. Stat. Methods Appl., 32, 237–270. https://doi.org/10.1007/s10260-022-00643-4
8. Alanis-Lobato, G., Cannistraci, C. V., Eriksson, A., Manica, A., & Ravasi, T. (2015). Highlighting nonlinear patterns in population genetics datasets. 1 Sci. Rep., 5, Article 8140. https://doi.org/10.1038/srep08140
9. Alipour, H., Muñoz, M. A., & Smith-Miles, K. (2023). Enhanced instance space analysis for the maximum flow problem. Eur. J. Oper. Res., 304(2), 411–428. https://doi.org/10.1016/j.ejor.2022.04.012
10. Alirezanejad, M., Enayatifar, R., Motameni, H., & Nematzadeh, H. (2020). Heuristic filter feature selection methods for medical datasets. Genomics, 112(2), 1173–1181. https://doi.org/10.1016/j.ygeno.2019.07.002
11. Altmann, A., Toloşi, L., Sander, O., & Lengauer, T. (2010). Permutation importance: a corrected feature importance measure. Bioinformatics, 26(10), 1340–1347. https://doi.org/10.1093/bioinformatics/btq134
12. Amado, C., Bianco, A. M., Boente, G., & Rodrigues, I. M. (2025). Robust estimation of heteroscedastic regression models: A brief overview and new proposals. Stat. Papers, 66, 65. https://doi.org/10.1007/s00362-025-01686-x
13. Ambroise, C., & McLachlan, G. J. (2002). Selection bias in gene extraction on the basis of microarray gene-expression data. Proc. Natl. Acad. Sci. USA, 99(10), 6562–6566. https://doi.org/10.1073/pnas.102102699
14. Amornbunchornvej, C., Zheleva, E., & Berger-Wolf, T. (2021). Variable-lag Granger causality and transfer entropy for time series analysis. ACM Trans. Knowl. Discov. Data, 15(4), 67. https://doi.org/10.1145/3441452
15. Anandhi, P., & Nathiya, E. (2023). Application of linear regression with their advantages, disadvantages, assumption and limitations. Int. J. Stat. Appl. Math., 8(6), 133-137. https://doi.org/10.22271/maths.2023.v8.i6b.1463
16. Aniceto, N., Bonifácio, V. D. B., Guedes, R. C., & Martinho, N. (2022). Exploring the Chemical Space of Urease Inhibitors to Extract Meaningful Trends and Drivers of Activity. J. Chem. Inf. Model., 62(15), 3535–3550. https://doi.org/10.1021/acs.jcim.2c00150
17. Ardelean, E.-R., Portase, R. L., Potolea, R., & Dînșoreanu, M. (2025). A path-based distance computation for non-convexity with applications in clustering. Knowl. Inf. Syst., 67, 1415–1453. https://doi.org/10.1007/s10115-024-02275-4
18. Asensio, J. O., Verheijen, M., & Caiment, F. (2022). Predicting missing proteomics values using machine learning: Filling the gap using transcriptomics and other biological features. Comput. Struct. Biotechnol. J., 20, 2057–2069. https://doi.org/10.1016/j.csbj.2022.04.017
19. Attia, Z. I., Lerman, G., & Friedman, P. A. (2021). Deep neural networks learn by using human-selected electrocardiogram features and novel features. Eur. Heart J. - Digit. Health, 2(3), 446–455. https://doi.org/10.1093/ehjdh/ztab060
20. Azim, R., Wang, S., & Dipu, S. A. (2022). CDSImpute: An ensemble similarity imputation method for single-cell RNA sequence dropouts. Comput. Biol. Med., 146, 105658. https://doi.org/10.1016/j.compbiomed.2022.105658
21. Banerjee, I., Bhattacharjee, K., Burns, J. L., Trivedi, H., Purkayastha, S., Seyyed-Kalantari, L., Patel, B. N., Shiradkar, R., & Gichoya, J. (2023). “Shortcuts” Causing Bias in Radiology Artificial Intelligence: Causes, Evaluation, and Mitigation. J. Am. Coll. Radiol., 20(9), 842–851. https://doi.org/10.1016/j.jacr.2023.06.025
22. Bansal, S., & Singh, G. (2023). Multiple linear regression based analysis of weather data: Assumptions and limitations. In R. N. Shaw, M. Paprzycki, & A. Ghosh (Eds.), Adv. Commun. Intell. Syst. ICACIS 2023 (Vol. 1920, pp. 169-178). Springer, Cham. https://doi.org/10.1007/978-3-031-45121-8_19
23. Barton-Henry, K., Wenz, L., & Levermann, A. (2021). Decay radius of climate decision for solar panels in the city of Fresno, USA. Sci. Rep., 11(1), 8571. https://doi.org/10.1038/s41598-021-87714-w
24. Belz, J., Nelles, O., Schwingshackl, D., Rehrl, J., & Horn, M. (2017). Order Determination and Input Selection with Local Model Networks. IFAC-PapersOnLine, 50(1), 7327–7332. https://doi.org/10.1016/j.ifacol.2017.08.1475
25. Ben-Naim, A. (2023). Intermolecular Interactions, Correlations, and Mutual Information. In Information Theory and Selected Applications (pp. 19-38). Springer, Cham. https://doi.org/10.1007/978-3-031-21276-5_2
26. Betz, J. L., & Sadler, J. R. (1981). Variants of a cloned synthetic lactose operator II. Chloramphenicol-resistant revertants retaining a lactose operator in the CAT gene of plasmid pBR325. Gene, 15(2-3), 187–200. https://doi.org/10.1016/0378-1119(81)90128-1
27. Bickel, D. R. (2008). Correcting the estimated level of differential expression for gene selection bias: application to a microarray study. Statistical Applications in Genetics and Molecular Biology, 7(1), Article10. https://doi.org/10.2202/1544-6115.1330
28. Bilodeau, B., Jaques, N., Koh, P. W., & Kim, B. (2024). Impossibility theorems for feature attribution. Proc. Natl. Acad. Sci., 121(2), e2304406120. https://doi.org/10.1073/pnas.2304406120
29. Black, D., Byrne, D., Walke, A., Choi, C., Buch, V., Wu, C.-C., Golby, A. J., & Pieper, S. (2024). Towards machine learning-based quantitative hyperspectral image guidance for brain tumor resection. Commun. Med., 4(1), Article 131. https://doi.org/10.1038/s43856-024-00562-3
30. Bobrowski, L. (1991). Design of piecewise linear classifiers from formal neurons by a basis exchange technique. Pattern Recognit., 24(9), 863–870. https://doi.org/10.1016/0031-3203(91)90005-P
31. Bobrowski, L., & Niemiro, W. (1984). A method of synthesis of linear discriminant function in the case of nonseparability. Pattern Recognit., 17(2), 205–210. https://doi.org/10.1016/0031-3203(84)90059-1
32. Bougioukos, P., Glotsos, D., Cavouras, D., Daskalakis, A., Kalatzis, I., Kostopoulos, S., Nikiforidis, G., & Bezerianos, A. (2010). An intensity-region driven multi-classifier scheme for improving the classification accuracy of proteomic MS-spectra. Comput. Methods Programs Biomed., 99(2), 147–153. https://doi.org/10.1016/j.cmpb.2009.11.003
33. Brinkrolf, J., Göpfert, C., & Hammer, B. (2019). Differential privacy for learning vector quantization. Neurocomputing, 342, 125–136. https://doi.org/10.1016/j.neucom.2018.11.095
34. Burgard, J. P., Moreira Costa, C., & Schmidt, M. (2024). Robustification of the k-means clustering problem and tailored decomposition methods: When more conservative means more accurate. Ann. Oper. Res., 339, 1525–1568. https://doi.org/10.1007/s10479-022-04818-w
35. Bushra, A. A., Kim, D., Kan, Y., & Yi, G. (2024). AutoSCAN: Automatic detection of DBSCAN parameters and efficient clustering of data in overlapping density regions. PeerJ Computer Science, 10, e1921. https://doi.org/10.7717/peerj-cs.1921
36. Cai, Y., Wu, S., Zhao, W., Li, Z., Wu, Z., & Ji, S. (2018). Concussion classification via deep learning using whole-brain white matter fiber strains. PLoS ONE, 13(5), e0197992. https://doi.org/10.1371/journal.pone.0197992
37. Caprihan, A., Pearlson, G. D., & Calhoun, V. D. (2008). Application of principal component analysis to distinguish patients with schizophrenia from healthy controls based on fractional anisotropy measurements. Neuroimage, 42(2), 675-682. https://doi.org/10.1016/j.neuroimage.2008.04.255
38. Carletti, M., Terzi, M., & Susto, G. A. (2023). Interpretable Anomaly Detection with DIFFI: Depth-based feature importance of Isolation Forest. Eng. Appl. Artif. Intell., 119, 105730. https://doi.org/10.1016/j.engappai.2022.105730
39. Caserini, N. A., & Pagnottoni, P. (2022). Effective transfer entropy to measure information flows in credit markets. Stat. Methods Appl., 31, 729–757. https://doi.org/10.1007/s10260-021-00614-1
40. Castaldi, P. J., Dahabreh, I. J., & Ioannidis, J. P. A. (2011). An empirical assessment of validation practices for molecular classifiers. Briefings in Bioinformatics, 12(3), 189–202. https://doi.org/10.1093/bib/bbq073
41. Cechinel, M. A. P., Neves, J., Fuck, J. V. R., de Andrade, R. C., Spogis, N., Riella, H. G., Padoin, N., & Soares, C. (2024). Enhancing wastewater treatment efficiency through machine learning-driven effluent quality prediction: A plant-level analysis. J. Water Process Eng., 58, 104758. https://doi.org/10.1016/j.jwpe.2023.104758
42. Cerqueti, R., Mattera, R., & Ficcadenti, V. (2024). Kendall correlations and radar charts to include goals for and goals against in soccer rankings. Comput. Stat.. Advance online publication. https://doi.org/10.1007/s00180-024-01542-w
43. Chanderraj, R., Brown, C. A., Hinkle, K., Falkowski, N., Woods, R. J., & Dickson, R. P. (2022). The bacterial density of clinical rectal swabs is highly variable, correlates with sequencing contamination, and predicts patient risk of extraintestinal infection. Microbiome, 10(1), 2. https://doi.org/10.1186/s40168-021-01190-y
44. Chanderraj, R., Brown, C. A., Hinkle, K., Falkowski, N., Woods, R. J., Dickson, R. P., & McMahon, K. (2020). Gut Microbiota Predict Enterococcus Expansion but Not Vancomycin-Resistant Enterococcus Acquisition. mSphere, 5(6), e00537-20. https://doi.org/10.1128/mSphere.00537-20
45. Chaudhari, M., Thapa, N., Roy, K., Newman, R. H., Saigo, H., & B K C, D. (2020). DeepRMethylSite: a deep learning based approach for prediction of arginine methylation sites in proteins. Mol. Omics, 16(5), 448–454. https://doi.org/10.1039/d0mo00025f
46. Chen, H., Mao, P., Lu, Y., & Rao, Y. (2023). Nonlinear Structural Equation Model Guided Gaussian Mixture Hierarchical Topic Modeling. In Proc. 61st Annu. Meet. Assoc. Comput. Linguist. (Vol. 1 Long Pap.) (pp. 10377-10390). Association for Computational Linguistics. https://doi.org/10.18653/v1/2023.acl-long.578
47. Chen, J., Qi, L., Wei, T., Zhang, S., Li, J., Asplund, C. L., Eickhoff, S. B., Bzdok, D., Holmes, A. J., & Yeo, B. T. T. (2023). Relationship between prediction accuracy and feature importance reliability: An empirical and theoretical study. Neuroimage, 274, 120115–120115. https://doi.org/10.1016/j.neuroimage.2023.120115
48. Chen, M., Papadikis, K., Jun, C., & Macdonald, N. (2023). Linear, nonlinear, parametric, and nonparametric regression models for nonstationary flood frequency analysis. J. Hydrol., 616, 128772. https://doi.org/10.1016/j.jhydrol.2022.128772
49. Chen, S., Ghadami, A., & Epureanu, B. I. (2022). Practical guide to using Kendall's τ in the context of forecasting critical transitions. R. Soc. Open Sci., 9(7), 211346. https://doi.org/10.1098/rsos.211346
50. Chen, V., Yang, M., Cui, W., Kim, J. S., Talwalkar, A., & Ma, J. (2024). Applying interpretable machine learning in computational biology—pitfalls, recommendations and opportunities for new developments. 2 Nature Methods, 21(8), 1454–1461. https://doi.org/10.1038/s41592-024-02359-7
51. Chen, Y. T., & Witten, D. M. (2023). Selective inference for k-means clustering. J. Mach. Learn. Res., 24, 152. https://doi.org/10.48550/arXiv.2203.15267
52. Chen, Y., Clayton, E. W., Novak, L. L., Anders, S., & Malin, B. (2023). Human-Centered Design to Address Biases in Artificial Intelligence. J. Med. Internet Res., 25, e43251. https://doi.org/10.2196/43251
53. Cheng, D., Zhang, C., Li, Y., Xia, S., Wang, G., Huang, J., Zhang, S., & Xie, J. (2024). GB-DBSCAN: A fast granular-ball based DBSCAN clustering algorithm. Inf. Sci., 674, 120731. https://doi.org/10.1016/j.ins.2024.120731
54. Cheungpasitporn, W., Thongprayoon, C., & Kashani, K. B. (2024). Artificial intelligence and machine learning’s role in sepsis-associated acute kidney injury. Kidney Res. Clin. Pract., 43(4), 417–432. https://doi.org/10.23876/j.krcp.23.298
55. Chow, C. F. W., Ghosh, S., Hadarovich, A., & Toth-Petroczy, A. (2024). SHARK enables sensitive detection of evolutionary homologs and functional analogs in unalignable and disordered sequences. Proc. Natl. Acad. Sci., 121(42), e2401622121. https://doi.org/10.1073/pnas.2401622121
56. Coronado, S., Martinez, J. N., Gualajara, V., & Rojas, O. (2022). Transfer entropy Granger causality between news indices and stock markets in U.S. and Latin America during the COVID-19 pandemic. Entropy, 24(10), 1420. https://doi.org/10.3390/e24101420
57. Costes, V., Sellem, E., Marthey, S., Tosser-Klopp, G., Klopp, C., & Elis, S. (2024). Multi-omics data integration for the identification of biomarkers for bull fertility. PLoS ONE, 19(2), e0298623. https://doi.org/10.1371/journal.pone.0298623
58. Cristian, P. M., Aarón, V. J., Armando, E. D., Jesús, E. S. V., & José, L. G. H. (2024). Diffusion on PCA-UMAP manifold: The impact of data structure preservation to denoise high-dimensional single-cell RNA sequencing data. Biology, 13(7), Article 512. https://doi.org/10.3390/biology13070512
59. Cummings, M. J., Bakamutumaho, B., Jain, K., Ssebambulidde, K., Ssekasanvu, J., Nakanjako, D., Akech, S., & Consortium, C. A. R. I. (2023). Development of a Novel Clinicomolecular Risk Index to Enhance Mortality Prediction and Immunological Stratification of Adults Hospitalized with Sepsis in Sub-Saharan Africa: A Pilot Study from Uganda. The American Journal of Tropical Medicine and Hygiene, 108(3), 619–626. https://doi.org/10.4269/ajtmh.22-0483
60. Cun, Y., & Fröhlich, H. (2012). Biomarker gene signature discovery integrating network knowledge. Biology, 1(1), 5–17. https://doi.org/10.3390/biology1010005
61. Dai, H., & Bao, Y. (2009). An inverse probability 3 weighted estimator for the bivariate distribution function under right censoring. Stat. Probab. Lett., 79(16), 1789–1797. https://doi.org/10.1016/j.spl.2009.05.010
62. Dankwa-Mullan, I. (2024). Health Equity and Ethical Considerations in Using Artificial Intelligence in Public Health and Medicine. Preventing Chronic Disease, 21, E64. https://doi.org/10.5888/pcd21.240245
63. Debray, M. P., Borie, R., Revel, M. P., Naccache, J. M., Khalil, A., Toper, C., Israel-Biet, D., Estellat, C., & Brillet, P. Y. (2015). Interstitial lung disease in anti-synthetase syndrome: Initial and follow-up CT findings. Eur. J. Radiol., 84(3), 516–523. https://doi.org/10.1016/j.ejrad.2014.11.026
64. Demircioğlu, A. (2021). Measuring the bias of incorrect application of feature selection when using cross-validation in radiomics. Insights Imaging, 12(1), 172. https://doi.org/10.1186/s13244-021-01115-1
65. Deng, Y., Hung, K. S. Y., Lui, S. S. Y., Lee, J. C. W., Wang, Y., Li, Z., Mak, H. K. F., Sham, P. C., Chan, R. C. K., & Cheung, E. F. C. (2019). Tractography-based classification in distinguishing patients with first-episode schizophrenia from healthy individuals. Progress in Neuro-Psychopharmacology and Biological Psychiatry, 88, 66–73. https://doi.org/10.1016/j.pnpbp.2018.06.010
66. Derrac, J., García, S., Molina, D., & Herrera, F. (2011). A practical tutorial on the use of nonparametric statistical tests as a methodology for comparing evolutionary and swarm intelligence algorithms. Swarm Evol. Comput., 1(1), 3-18. https://doi.org/10.1016/j.swevo.2011.02.002
67. Dey, D., Haque, M. S., Islam, M. M., Aishi, U. I., Shammy, S. S., Mayen, M. S. A., Noor, S. T. A., & Uddin, M. J. (2025). The proper application of logistic regression model in complex survey data: a systematic review. BMC Med. Res. Methodol., 25, 15. https://doi.org/10.1186/s12874-024-02454-5
68. Dey, R., & Lee, S. (2019). Asymptotic properties of principal component analysis and shrinkage-bias adjustment under the generalized spiked population model. J. Multivar. Anal., 173, 145-164. https://doi.org/10.1016/j.jmva.2019.02.007
69. Diaz, M., Moetesum, M., Siddiqi, I., & Vessio, 5 G. (2021). Sequence-based dynamic handwriting analysis for Parkinson’s disease detection with one-dimensional convolutions and BiGRUs. Expert Syst. Appl., 168, 114405. https://doi.org/10.1016/j.eswa.2020.114405
70. Ding, S., Shi, Z., & Azar, A. T. (2015). Research and development of advanced computing technologies. Sci. World J., 2015, 239723. https://doi.org/10.1155/2015/239723 6
71. Domingue, B. W., Kanopka, K., Trejo, S., Rhemtulla, M., & Tucker-Drob, E. M. (2024). Ubiquitous bias and false discovery due to model misspecification in analysis of statistical interactions: The role of the outcome's distribution and metric properties. Psychol. Methods, 29(6), 1164–1179. https://doi.org/10.1037/met0000532
72. Dong, M., Li, L., Chen, M., Kusalik, A., & Xu, W. (2020). Predictive analysis methods for human microbiome data with application to Parkinson’s disease. PLoS ONE, 15(8), e0237779. https://doi.org/10.1371/journal.pone.0237779
73. Donmez, B., McDonald, A. D., Lee, J. D., & Boyle, L. N. (2023). Road user behavior: Describing, inferring, predicting and beyond. Transp. Res. Interdiscip. Perspect., 22, 100932. https://doi.org/10.1016/j.trip.2023.100932 7
74. Dukart, J., Mueller, K., Barthel, H., Villringer, A., Sabri, O., & Schroeter, M. L. (2013). Meta-analysis based SVM classification enables accurate detection of Alzheimer’s disease across different clinical centers using FDG-PET and MRI. Psychiatry Res.: Neuroimaging, 212(3), 230–236. https://doi.org/10.1016/j.pscychresns.2012.04.007
75. Dunne, R., Reguant, R., Ramarao-Milne, P., Szul, P., Sng, L. M. F., Lundberg, M., Twine, N. A., & Bauer, D. C. (2023). Thresholding Gini variable importance with a single-trained random forest: An empirical Bayes approach. Comput. Struct. Biotechnol. J., 21, 4354-4360. https://doi.org/10.1016/j.csbj.2023.08.033
76. Dupeu, J. M. (1997). La consultation thérapeutique en pédo-psychiatrie: 10e journée annuelle de psychiatrie infantile. Journal de Pédiatrie et de Puériculture, 10(6), 349–355. https://doi.org/10.1016/S0987-7983(97)80099-0 8
77. Dyer, E. L., & Kording, K. (2023). Why the simplest explanation isn't always the best. Proc. Natl. Acad. Sci., 120(52), Article e2319169120. https://doi.org/10.1073/pnas.2319169120
78. Eden, S. K., Li, C., & Shepherd, B. E. (2022). Nonparametric estimation of Spearman's rank correlation with bivariate survival data. Biometrics, 78(2), 421-434. https://doi.org/10.1111/biom.13453
79. Effrosynidis, D., & Arampatzis, A. (2021). An evaluation of feature selection methods for environmental data. Ecol. Inform., 61, 101224. https://doi.org/10.1016/j.ecoinf.2021.101224
80. Ehrlich, D. A., Schick-Poland, K., Makkeh, A., Lanfermann, F., Wollstadt, P., & Wibral, M. (2024). Partial information decomposition for continuous variables based on shared exclusions: Analytical formulation and estimation. Phys. Rev. E, 110(1), 014115. https://doi.org/10.1103/PhysRevE.110.014115
81. Eichele, T., Rachakonda, S., & Calhoun, V. (2009). EEGIFT: A toolbox for group temporal ICA of event-related EEG. Neuroimage, 47(Supplement 1), S101. https://doi.org/10.1016/S1053-8119(09)70872-9
82. Eisele, A. S., Tarbier, M., Dormann, A. A., Pelechano, V., & Suter, D. M. (2024). Gene-expression memory-based prediction of cell lineages from scRNA-seq datasets. Nat. Commun., 15(1), 2744. https://doi.org/10.1038/s41467-024-47158-y
83. Ekhlasi, A., Motie Nasrabadi, A., & Mohammadi, M. (2023). Improving transfer entropy and partial transfer entropy for relative detection of effective connectivity strength between time series. Commun. Nonlinear Sci. Numer. Simul., 126, 107449. https://doi.org/10.1016/j.cnsns.2023.107449
84. El Allali, A., Elhamraoui, Z., & Daoud, R. (2021). Machine learning applications in RNA modification sites prediction. Comput. Struct. Biotechnol. J., 19, 5510–5524. https://doi.org/10.1016/j.csbj.2021.09.025 9
85. Elhaik, E. (2022). Principal component analyses (PCA)-based findings in population genetic studies are highly biased and must be reevaluated. Sci. Rep., 12(1), Article 14683. https://doi.org/10.1038/s41598-022-14395-4
86. Erman, B. (2023). Mutual information analysis of mutation, nonlinearity, and triple interactions in proteins. Proteins, 91(1), 121–133. https://doi.org/10.1002/prot.26415
87. Fakhraei, S., Soltanian-Zadeh, H., & Fotouhi, F. (2014). Bias and stability of single variable classifiers for feature ranking and selection. Expert Syst. Appl., 41(15), 6945–6958. https://doi.org/10.1016/j.eswa.2014.05.007
88. Fallahpour, S., Lakvan, E. N., & Zadeh, M. H. (2017). Using an ensemble classifier based on sequential floating forward selection for financial distress prediction problem. J. Retail. Consum. Serv., 34, 159–167. https://doi.org/10.1016/j.jretconser.2016.10.002 12
89. Felippe, H., Battiston, F., & Kirkley, A. (2024). Network mutual information measures for graph similarity. Commun. Phys., 7, 335. https://doi.org/10.1038/s42005-024-01830-3
90. Fisher, A., Rudin, C., & Dominici, F. (2019). All models are wrong, but many are useful: Learning a variable's importance by studying an entire class of prediction models simultaneously. J. Mach. Learn. Res., 20, 177. https://doi.org/10.48550/arXiv.1801.01489
91. Fox, J., & Monette, G. (1992). Generalized Collinearity Diagnostics. Journal of the American Statistical Association, 87(417), 178–183. https://doi.org/10.1080/01621459.1992.10475190
92. Frénay, B., Doquire, G., & Verleysen, M. (2014). Estimating mutual information for feature selection in the presence of label noise. Comput. Stat. & Data Analysis, 71, 832–848. https://doi.org/10.1016/j.csda.2013.05.001
93. Fujita, A., Sato, J. R., Demasi, M. A., Sogayar, M. C., Ferreira, C. E., & Miyano, S. (2009). Comparing Pearson, Spearman, and Hoeffding's D measure for gene expression association analysis. Journal of Bioinformatics and Computational Biology, 7(4), 663–684. https://doi.org/10.1142/s0219720009004230
94. Galligan, M. C., Saldova, R., Campbell, M. P., Rudd, P. M., & Murphy, T. B. (2013). Greedy feature selection for glycan chromatography data with the generalized Dirichlet distribution. BMC Bioinformatics, 14, 155. https://doi.org/10.1186/1471-2105-14-155
95. Gandhudi, M., Alphonse, P. J. A., Fiore, U., & Gangadharan, G. R. (2024). Explainable hybrid quantum neural networks for analyzing the influence of tweets on stock price prediction. Comput. Electr. Eng., 118(Part A), 109302. https://doi.org/10.1016/j.compeleceng.2024.109302
96. Ge, L., Chen, Y., Yan, C., Wang, J., Wu, H., & Zhang, X. (2019). Study Progress of Radiomics With Machine Learning for Precision Medicine in Bladder Cancer Management. Front. Oncol., 9, 1296. https://doi.org/10.3389/fonc.2019.01296
97. Ge, R., Zhou, M., Luo, Y., Zhang, Z., Zhang, R., & Ning, K. (2016). McTwo: a two-step feature selection algorithm based on maximal information coefficient. BMC Bioinformatics, 17, 142. https://doi.org/10.1186/s12859-016-0990-0
98. Gibson, J. D. (2025). Entropy and Mutual Information. In Information Theoretic Principles for Agent Learning (pp. 15-33). Springer, Cham. https://doi.org/10.1007/978-3-031-65388-9_2
99. Gnambs, T. (2023). A brief note on the standard error of the Pearson correlation. Collabra: Psychol., 9(1). https://doi.org/10.1525/collabra.87615
100. Goldfarb, E. V., Scheinost, D., Fogelman, N., Seo, D., & Sinha, R. (2022). High-Risk Drinkers Engage Distinct Stress-Predictive Brain Networks. Biol. Psychiatry: Cogn. Neurosci. Neuroimaging, 7(8), 805–813. https://doi.org/10.1016/j.bpsc.2022.02.010
101. Grabowski, E., & Kuo, J. (2023). Comparing K-means and OPTICS clustering algorithms for identifying vowel categories. Proc. Linguist. Soc. Am., 8(1), 5488. https://doi.org/10.3765/plsa.v8i1.5488
102. Grover, D., Bauhoff, S., & Friedman, J. (2019). Using supervised learning to select audit targets in performance-based financing in health: An example from Zambia. 1 PLoS ONE, 14(1), e0211262. 2 https://doi.org/10.1371/journal.pone.0211262
103. Gu, Z., Jamison, K. W., Sabuncu, M. R., & Kuceyeski, A. (2024). Machine learning and neuroimaging: Understanding the human brain in health and disease. In Neuroimaging Methods Appl. Comput. Netw. Model. Neuroimaging Data (pp. 261–285). Academic Press. https://doi.org/10.1016/B978-0-443-13480-7.00010-7
104. Guan, W., Zhou, M., Hampton, C. Y., дела Вега, Ф. М., Cogdell, D. E., Urbauer, D. L., Фенольо, М. И., & Bast, R. C. (2009). Ovarian cancer detection from metabolomic liquid chromatography/mass spectrometry data by support 3 vector machines. BMC Bioinformatics, 10, 259. https://doi.org/10.1186/1471-2105-10-259
105. Guidotti, R., Del Gratta, C., Baldassarre, A., Romani, G. L., & Corbetta, M. (2015). Visual Learning Induces Changes in Resting-State fMRI Multivariate Pattern of 4 Information. J. Neurosci., 35(27), 9786–9798. https://doi.org/10.1523/JNEUROSCI.3920-14.2015
106. Guyader, J.-M., Huizinga, W., Poot, D. H. J., van Kranenburg, M., Uitterdijk, A., Niessen, W. J., & Klein, S. (2018). Groupwise image registration based on a total correlation dissimilarity measure for quantitative MRI and dynamic imaging data. Sci. Rep., 8, 13112. https://doi.org/10.1038/s41598-018-31474-7
107. Guyatt, G. H., Webber, C. E., Mewa, A. A., & Sackett, D. L. (1984). Determining causation—A case study: Adrenocorticosteroids and osteoporosis: Should the fear of inducing clinically important osteoporosis 5 influence the decision 6 to prescribe adrenocorticosteroids? J. Chronic Dis., 37(5), 343–352. https://doi.org/10.1016/0021-9681(84)90100-0
108. Hair, J. F., Hult, G. T. M., Ringle, C. M., Sarstedt, M., Danks, N. P., & Ray, S. (2021). An introduction to structural equation modeling. In Partial Least Squares Struct. Equ. Model. (PLS-SEM) Using R: Classr. Companion: Bus. (pp. 1-25). Springer. https://doi.org/10.1007/978-3-030-80519-7_1
109. Hair, J. F., Sarstedt, M., Ringle, C. M., Sharma, P. N., & Liengaard, B. D. (2024). Going beyond the untold facts in PLS–SEM and moving forward. Eur. J. Mark., 58(13), 81–106. https://doi.org/10.1108/EJM-08-2023-0645
110. Hajihosseinlou, A., Maghsoudi, A., & Ghezelbash, R. (2024). A comprehensive evaluation of OPTICS, GMM and K-means clustering methodologies for geochemical anomaly detection connected with sample catchment basins. Geochemistry, 84(2), Article 126094. https://doi.org/10.1016/j.chemer.2024.126094
111. Han, H. (2017). A novel feature selection for RNA-seq analysis. Computational Biology and Chemistry, 71, 245–257. https://doi.org/10.1016/j.compbiolchem.2017.10.010
112. Han, J., & Kang, S. (2021). Active learning with missing values considering imputation uncertainty. Knowl. Based Syst., 224, 107079. https://doi.org/10.1016/j.knosys.2021.107079
113. Hanbay, K. (2022). A new standard error based artificial bee colony algorithm and its applications in feature selection. Journal of King Saud University - Computer and Inf. Sci., 34(7), 4554–4567. https://doi.org/10.1016/j.jksuci.2021.04.010
114. Hancer, E., Xue, B., & Zhang, M. (2018). Differential evolution for filter feature selection based on information theory and feature ranking. Knowl. Based Syst., 140, 103–119. https://doi.org/10.1016/j.knosys.2017.10.028
115. Higham, P. A., & Higham, D. P. (2019). New improved gamma: Enhancing the accuracy of Goodman-Kruskal's gamma using ROC curves. Behav. Res. Methods, 51(1), 108–125. https://doi.org/10.3758/s13428-018-1125-5
116. Hooshyar, D., & Yang, Y. (2024). Problems With SHAP and LIME in Interpretable AI for Education: A Comparative Study of Post-Hoc Explanations and Neural-Symbolic Rule Extraction. IEEE Access, 12, 137472-137490. https://doi.org/10.1109/ACCESS.2024.3463948
117. Horrace, W. C., & Oaxaca, R. L. (2006). Results on the bias and inconsistency of ordinary least squares for the linear probability model. Econ. Lett., 90(3), 321-327. https://doi.org/10.1016/j.econlet.2005.08.024
118. Hou, J., Ye, X., Feng, W., Zhang, Q., Han, Y., Liu, Y., Li, Y., & Wei, Y. (2022). Distance correlation application to gene co-expression network analysis. BMC Bioinformatics, 23(1), 81. https://doi.org/10.1186/s12859-022-04609-x
119. Hsieh, M. H., Eisenberg, M. L., Hittelman, A. B., Wilson, J. M., Tasian, G. E., & Baskin, L. S. (2012). Caucasian male infants and boys with hypospadias exhibit reduced anogenital distance. Hum. Reprod., 27(6), 1577–1580. https://doi.org/10.1093/humrep/des087
120. Huang, J., Cai, Y., & Xu, X. (2007). A hybrid genetic algorithm for feature selection wrapper based on mutual information. Pattern Recognit. Letters, 28(13), 1 1825–1844. https://doi.org/10.1016/j.patrec.2007.05.011
121. Huang, X., & Marques-Silva, J. (2024). On the failings of Shapley values for explainability. Int. J. Approx. Reason., 171, 109112. https://doi.org/10.1016/j.ijar.2023.109112
122. Huang, X., Tao, H., Ni, H., & Hou, C. (2025). Debiasing weighted multi-view k-means clustering based on causal regularization. Pattern Recognit., 160, 111195. https://doi.org/10.1016/j.patcog.2024.111195
123. Humberg, S., Grund, S., & Nestler, S. (2024). Estimating nonlinear effects of random slopes: A comparison of multilevel structural equation modeling with a two-step, a single-indicator, and a plausible values approach. Behav. Res. Methods, 56(7), 7912–7938. https://doi.org/10.3758/s13428-024-02462-9
124. Huti, M., Lee, T., Sawyer, E., & King, A. P. (2023). An investigation into race bias in random forest models based on breast DCE-MRI derived radiomics features. In Clinical Image Based Procedure Fairness AI Med Imaging Ethical Philos Issues Med Imaging (Vol. 14242, pp. 225-234). https://doi.org/10.1007/978-3-031-45249-9_22
125. Irmer, J. P., Klein, A. G., & Schermelleh-Engel, K. (2024). Estimating power in complex nonlinear structural equation modeling including moderation effects: The powerNLSEM R-package. Behav. Res. Methods, 56, 8897-8931. https://doi.org/10.3758/s13428-024-02476-3
126. Jacob, J., & Varadharajan, R. (2024). Robust Variance Inflation Factor: A Promising Approach for Collinearity Diagnostics in the Presence of Outliers. Sankhya B, 86, 845–871. https://doi.org/10.1007/s13571-024-00342-y
127. Jain, R., & Xu, W. (2021). HDSI: High dimensional selection with interactions algorithm on feature selection and testing. PLoS ONE, 16(2), e0246159. https://doi.org/10.1371/journal.pone.0246159
128. Janse, R. J., Hoekstra, T., Jager, K. J., Zoccali, C., Tripepi, G., Dekker, F. W., & van Diepen, M. (2021). Conducting correlation analysis: Important limitations and pitfalls. Clin. Kidney J., 14(11), 2332-2337. https://doi.org/10.1093/ckj/sfab085
129. Jarantow, S. W., Pisors, E. D., & Chiu, M. L. (2023). Introduction to the use of linear and nonlinear regression analysis in quantitative biological assays. Curr. Protoc., 3(6), e801. https://doi.org/10.1002/cpz1.801
130. Jeung, M., Jang, M., Shin, K., Jung, S. W., & Baek, S. (2025). Graph neural networks and transfer entropy enhance forecasting of mesozooplankton community dynamics. Environ. Sci. Ecotechnol., 23, 100514. https://doi.org/10.1016/j.ese.2024.100514
131. Jha, A., Menozzi, E., Oyekan, R., двигатель, Д., Pajek, J., Prihodova, L., Růžičková, H., Růžička, F., Škoch, A., & господин, Т. (2020). The Cloud UPDRS smartphone software in Parkinson’s study: cross-validation against blinded human raters. NPJ Parkinsons Dis., 6(1), 36. https://doi.org/10.1038/s41531-020-00135-w
132. Jiang, J., Xiao, Y., Liu, J., Zhang, Y., Zhang, J., Wang, H., & Zhang, X. (2024). T1 mapping-based radiomics in the identification of histological types of lung cancer: a reproducibility and feasibility study. BMC Med. Imaging, 24(1), 308. https://doi.org/10.1186/s12880-024-01487-y
133. Jin, S., Noh, M., Yang-Wallentin, F., & Lee, Y. (2021). Robust nonlinear structural equation modeling with interaction between exogenous and endogenous latent variables. Struct. Equ. Model.: Multidiscip. J., 28(4), 547–556. https://doi.org/10.1080/10705511.2020.1857255
134. Jollans, L., Boyle, R., Artiges, E., Banaschewski, T., Barker, G. J., Büchel, C., Cattrell, A., Conrod, P. J., Desrivières, S., Flor, H., Frouin, V., Garavan, H., Gowland, P., Heinz, A., Ittermann, B., Lemaître, H., Martinot, J.-L., Nees, F., Orfanos, D. P., … Schumann, G. (2019). Quantifying performance of machine learning methods for neuroimaging data. Neuroimage, 199, 351–365. https://doi.org/10.1016/j.neuroimage.2019.05.082
135. Jung, W. S., Lee, J. S., Solander, S., & Choi, J. W. (2020). Pseudo-Occlusion of the Internal Carotid Artery in Acute Ischemic Stroke: Clinical Outcome 2 after Mechanical Thrombectomy. Sci. Rep., 10(1), 2832. https://doi.org/10.1038/s41598-020-59609-9
136. Junges, R., Lomazzi, L., Miele, L., Giglio, M., & Cadini, F. (2024). Mitigating the Impact of Temperature Variations on Ultrasonic Guided Wave-Based Structural Health Monitoring 3 through Variational Autoencoders. Sensors, 24(5), 1494. https://doi.org/10.3390/s24051494
137. Kakuba, S., & Han, D. S. (2024). Addressing data scarcity in speech emotion recognition: A comprehensive review. ICT Express. https://doi.org/10.1016/j.icte.2024.11.003
138. Kamat, P., Kumar, S., & Kotecha, K. (2024). DeepTool: A deep learning framework for tool wear onset detection and remaining useful life prediction. MethodsX, 13, 102965. https://doi.org/10.1016/j.mex.2024.102965
139. Katal, S., York, B., & Gholamrezanezhad, A. (2024). AI in radiology: From promise to practice − A guide to effective integration. Eur. J. Radiol., 181, 111798. https://doi.org/10.1016/j.ejrad.2024.111798
140. Kazienko, P., & Kajdanowicz, T. (2012). Label-dependent node classification in the network. Neurocomputing, 75(1), 199–209. https://doi.org/10.1016/j.neucom.2011.04.047
141. Kepplinger, D. (2023). Robust variable selection and estimation via adaptive elastic net S-estimators for linear regression. Comput. Stat. & Data Analysis, 183, 107730. https://doi.org/10.1016/j.csda.2023.107730
142. Kerby, T., White, T., & Moon, K. R. (2024). Learning local higher-order interactions with total correlation. In Proc. 2024 IEEE 34th Int. Workshop Mach. Learn. Signal Process. (MLSP) (pp. 1-6). IEEE. https://doi.org/10.1109/MLSP58920.2024.10734758
143. Keshavan, M. S., Collin, G., Guimond, S., Kelly, S., Prasad, K. M., 4 & Lizano, P. (2020). Neuroimaging in Schizophrenia. Neuroimaging Clin. N. Am., 30(1), 73–83. https://doi.org/10.1016/j.nic.2019.09.007
144. Khan, I. K., Daud, H. B., Zainuddin, N. B., Sokkalingam, R., Farooq, M., Baig, M. E., Ayub, G., & Zafar, M. (2024). Determining the optimal number of clusters by Enhanced Gap Statistic in K-mean algorithm. Egypt. Inform. J., 27, 100504. https://doi.org/10.1016/j.eij.2024.100504
145. Kipruto, E., & Sauerbrei, W. (2024). Post-Estimation Shrinkage in Full and Selected Linear Regression Models in Low-Dimensional Data Revisited. Biometrical Journal. Biometrische Zeitschrift, 66(7), e202300368. https://doi.org/10.1002/bimj.202300368
146. Kiviet, J. F., & Phillips, G. D. A. (1996). The bias of the ordinary least squares estimator in simultaneous equation models. Econ. Lett., 53(2), 161-167. https://doi.org/10.1016/S0165-1765(96)00908-1
147. Krawczuk, J., & Łukaszuk, T. (2016). The feature selection bias problem in relation to high-dimensional gene data. Artif. Intell. Med., 66, 63-71. https://doi.org/10.1016/j.artmed.2015.11.001
148. Kretowska, M. (2018). Tree-based models for survival data with competing risks. Comput. Methods Programs Biomed., 159, 185–198. https://doi.org/10.1016/j.cmpb.2018.03.017
149. Ku, W. L., & Min, H. (2024). Evaluating Machine Learning Stability in Predicting Depression and Anxiety Amidst Subjective Response Errors. Healthcare (Basel, Switz.), 12(6), 625. https://doi.org/10.3390/healthcare12060625
150. Kumar, A. (2024). Comparative evaluation of linear and nonlinear regression models in predicting VLC channel response and BER performance. J. Opt.. https://doi.org/10.1007/s12596-024-02361-4
151. Kumar, I., Scheidegger, C., Venkatasubramanian, S., & Friedler, S. (2021). Shapley residuals: Quantifying the limits of the Shapley value for explanations. Adv. Neural Inf. Process. Syst., 34, 26598–26608.
152. Kunert-Graf, J., Sakhanenko, N., & Galas, D. (2020). Partial Information Decomposition and the Information Delta: A Geometric Unification Disentangling Non-Pairwise Information. Entropy (Basel, Switzerland), 22(12), 1333. https://doi.org/10.3390/e22121333
153. Kursa, M. B. (2022). Kendall transformation brings a robust categorical representation of ordinal data. Sci. Rep., 12, 8341. https://doi.org/10.1038/s41598-022-12224-2
154. Lee, T., Kim, Y., Hyun, Y., Mo, J., & Yoo, Y. (2024). Unsupervised anomaly detection process using LLE and HDBSCAN by Style-GAN as a feature extractor. Int. J. Precis. Eng. Manuf., 25, 51–63. https://doi.org/10.1007/s12541-023-00908-2
155. Lenhof, K., Eckhart, L., Rolli, L. M., & Lenhof, H. P. (2024). Trust me if you can: A survey on reliability and interpretability of machine learning approaches for drug sensitivity prediction in cancer. Briefings in Bioinformatics, 25(5), bbae379. https://doi.org/10.1093/bib/bbae379
156. Lenz, M., Müller, F. J., Zenke, M., & Schuppert, A. (2016). Principal components analysis and the reported low intrinsic dimensionality of gene expression microarray data. Sci. Rep., 6, Article 25696. https://doi.org/10.1038/srep25696
157. Li, L., Kang, Y., & Li, F. (2023). Bayesian forecast combination using time-varying features. Int. J. Forecast., 39(3), 1287–1302. https://doi.org/10.1016/j.ijforecast.2022.06.002 7
158. Li, Q., Ver Steeg, G., & Malo, J. (2024). Functional connectivity via total correlation: Analytical results in visual areas. Neurocomputing, 571, 127143. https://doi.org/10.1016/j.neucom.2023.127143
159. Li, W. X., Lin, Q. H., Zhang, C. Y., Han, Y., & Calhoun, V. D. (2024). A new transfer entropy method for measuring directed connectivity from complex-valued fMRI data. Front. Neurosci., 18, 1423014. https://doi.org/10.3389/fnins.2024.1423014
160. Li, X., Shao, B., & Bian, G. (2024). A scholars’ personality traits augmented multi-dimensional feature fusion scholarly journal recommendation model. Appl. Soft Comput., 163, 111888. https://doi.org/10.1016/j.asoc.2024.111888 8
161. Li, Y., Liang, M., Mao, L., & Wang, S. (2021). Robust estimation and variable selection for the accelerated failure time model. Stat. Med., 40(20), 4473-4491. https://doi.org/10.1002/sim.9042
162. Liang, J. E. (2024). Partial information decomposition for causal discovery with application to Internet of Things. IEEE Internet Things J., 11(13), 24289-24299. https://doi.org/10.1109/JIOT.2024.3390449
163. Lin, H., Eggesbø, M., & Peddada, S. D. (2022). Linear and nonlinear correlation estimators unveil undescribed taxa interactions in microbiome data. Nature Communications, 13, 4946. https://doi.org/10.1038/s41467-022-32243-x
164. Lin, H., Zou, W., Li, T., Feigenberg, S. J., Teo, B. K., & Dong, L. (2019). A Super-Learner Model for Tumor Motion Prediction and Management in Radiation Therapy: Development and Feasibility Evaluation. Sci. Rep., 9(1), 14868. https://doi.org/10.1038/s41598-019-51338-y 9
165. Linardatos, P., Papastefanopoulos, V., & Kotsiantis, S. (2020). Explainable AI: A Review of Machine Learning Interpretability Methods. Entropy, 23(1), 18. https://doi.org/10.3390/e23010018
166. Lipton, Z. C. (2018). The mythos of model interpretability: In machine learning, the concept of interpretability is both important and slippery. Queue, 16(3), 31–57. https://doi.org/10.1145/3236386.3241340
167. Liu, H., Yuan, H., Wang, Y., Huang, W., Xue, H., & Zhang, X. (2021). Prediction of venous thromboembolism with machine learning techniques in young-middle-aged inpatients. Sci. Rep., 11(1), 12868. https://doi.org/10.1038/s41598-021-92287-9
168. Liu, J. (2025). Examination of nonlinear longitudinal processes with latent variables, latent processes, latent changes, and latent classes in the structural equation modeling framework: The R package nlpsem. Behav. Res. Methods, 57(3), 87. https://doi.org/10.3758/s13428-025-02596-4
169. Liu, T., Wang, M., Yang, B., Liu, H., & Yi, S. (2025). ESERNet: Learning spectrogram structure relationship for effective speech emotion recognition with swin transformer in classroom discourse analysis. Neurocomputing, 612, 128711. https://doi.org/10.1016/j.neucom.2024.128711
170. Liu, X., Zhou, Y., & Zongrun, W. (2020). Can the development of a patient’s condition be predicted through intelligent inquiry under the e-health business mode? Sequential feature map-based disease risk prediction upon features selected from cognitive diagnosis big data. Int. J. Inf. Manag., 50, 463–486. https://doi.org/10.1016/j.ijinfomgt.2019.05.006
171. Liu, Y., Yuan, X., Jiang, X., Wang, P., Kou, J., Wang, H., & Liu, M. (2021). Dilated Adversarial U-Net Network for automatic gross tumor volume segmentation of nasopharyngeal carcinoma. Appl. Soft Comput., 111, 107722. https://doi.org/10.1016/j.asoc.2021.107722
172. Liu, Z., Chang, B., & Cheng, F. (2021). An interactive filter-wrapper multi-objective evolutionary algorithm for feature selection. Swarm Evol. Comput., 65, 100925. https://doi.org/10.1016/j.swevo.2021.100925
173. Loecher, M. (2024). Debiasing SHAP scores in random forests. AStA Advances in Statistical Analysis, 108, 427-440. https://doi.org/10.1007/s10182-023-00479-7
174. Lohrmann, C., & Luukka, P. (2022). Nonspecificity, strife and total uncertainty in supervised feature selection. Eng. Appl. Artif. Intell., 109, 104628. https://doi.org/10.1016/j.engappai.2021.104628
175. Lones, M. A. (2024). Avoiding common machine learning pitfalls. Patterns, 5(10), 101046. https://doi.org/10.1016/j.patter.2024.101046
176. Lovatti, B. P. O., Nascimento, M. H. C., Neto, A. C., Castro, E. V. R., & Filgueiras, P. R. (2019). Use of Random forest in the identification of important variables. Microchem. J., 145, 1129–1134. https://doi.org/10.1016/j.microc.2018.12.028
177. Lu, X., Qian, W., Dai, S., & Huang, J. (2024). Label distribution feature selection based on hierarchical structure and neighborhood granularity. Inf. Fusion, 112, 102588. https://doi.org/10.1016/j.inffus.2024.102588
178. Ma, C., Zhang, P., Pan, L., Li, X., Yin, C., Li, A., Zong, R., & Zhang, Z. (2022). A feature fusion sequence learning approach for quantitative analysis of tremor symptoms based on digital handwriting. Expert Syst. Appl., 203, 117400. https://doi.org/10.1016/j.eswa.2022.117400
179. Ma, H., Prosperino, D., Haluszczynski, A., & Räth, C. (2024). Linear and nonlinear causality in financial markets. Chaos, 34(11), 113125. https://doi.org/10.1063/5.0184267
180. Maggipinto, T., Bellotti, R., Amoroso, N., прав, М., водитель, П., Tangaro, S., Савио, С., & Jirsa, V. K. (2017). DTI measurements for Alzheimer’s classification. Phys. Med. Biol., 62(6), 2361–2375. https://doi.org/10.1088/1361-6560/aa5dbe
181. Mandler, H., & Weigand, B. (2024). A review and benchmark of feature importance methods for neural networks. ACM Comput. Surv., 56(12), 318. https://doi.org/10.1145/3679012
182. Manolov, R. (2023). Does the choice of a linear trend-assessment technique matter in the context of single-case data? Behav. Res. Methods, 55(8), 4200–4221. https://doi.org/10.3758/s13428-022-02013-0
183. Markowetz, F., & Spang, R. (2005). Molecular diagnosis. Classification, model selection and performance evaluation. Methods Inf. Med., 44(3), 438–443. https://doi.org/10.1055/s-0038-1633990
184. Mateo, F., Garcés-Iniesta, J. J., Jenninger, B., Gómez-Sanchís, J., Soria-Olivas, E., & Chiggiato, P. (2021). Automatic mass spectra recognition for Ultra High Vacuum systems using multilabel classification. Expert Syst. Appl., 178, 114959. https://doi.org/10.1016/j.eswa.2021.114959
185. Matusik, E., Vassal, O., Conrad, A., Ferry, T., Millet, A., Dupont, D., Grandjean, L., Guitton, J., Roux, S., Bienvenu, A.-L., Bohé, J., Friggeri, A., & Goutelle, S. (2024). Parametric and nonparametric population pharmacokinetic analysis of fluconazole in critically ill patients and dosing simulations for Candida infections. Antimicrob. Agents Chemother., 68(11), e0099124. https://doi.org/10.1128/aac.00991-24
186. Mazumder, P., & Singh, P. (2022). Protected attribute guided representation learning for bias mitigation in limited data. Knowl. Based Syst., 244, 108449. https://doi.org/10.1016/j.knosys.2022.108449
187. McLatchie, Y., & Vehtari, A. (2024). Efficient estimation and correction of selection-induced bias with order statistics. Stat. Comput., 34, 132. https://doi.org/10.1007/s11222-024-10442-4
188. Medjek, F., Tandjaoui, D., Djedjig, N., & Romdhani, I. (2021). Fault-tolerant AI-driven Intrusion Detection System for the Internet of Things. Int. J. Crit. Infrastruct. Prot., 34, 100436. https://doi.org/10.1016/j.ijcip.2021.100436
189. Mehta, P., Bukov, M., Wang, C.-H., Day, A. G. R., Richardson, C., Fisher, C. K., & Schwab, D. J. (2019). A high-bias, low-variance introduction to machine learning for physicists. Phys. Rep., 810, 1-124. https://doi.org/10.1016/j.physrep.2019.03.001
190. Merino-Soto, C., Juárez-García, A., Escudero, G. S., & Toledano-Toledano, F. (2022). Parametric and nonparametric analysis of the internal structure of the psychosocial work processes questionnaire (PROPSIT) as applied to workers. International Journal of Environ. Res. and Public Health, 19(13), 7970. https://doi.org/10.3390/ijerph19137970
191. Metsämuuronen, J. (2021). Directional nature of Goodman–Kruskal gamma and some consequences: Identity of Goodman–Kruskal gamma and Somers delta, and their connection to Jonckheere–Terpstra test statistic. Behaviormetrika, 48, 283–307. https://doi.org/10.1007/s41237-021-00138-8
192. Mieth, B., Rozier, A., Rodriguez, J. A., Höhne, M. M. C., Görnitz, N., & Müller, K.-R. (2021). DeepCOMBI: explainable artificial intelligence for the analysis and discovery in genome-wide association studies. NAR Genomics and Bioinformatics, 3(3), lqab065. https://doi.org/10.1093/nargab/lqab065
193. Mishra, A. K., & Das, B. (2024). A Hoeffding D statistic approach for detecting electricity theft. In Proc. 2024 IEEE 4th Int. Conf. Sustain. Energy Futur. Electr. Transp. (SEFET) (pp. 1–6). Hyderabad, India. https://doi.org/10.1109/SEFET61574.2024.10718232
194. Mohseni, N., & Elhaik, E. (2024). Biases of Principal Component Analysis (PCA) in Physical Anthropology Studies Require a Reevaluation of Evolutionary Insights. eLife, 13, RP94685. https://doi.org/10.7554/eLife.94685.2
195. Molnar, C., König, G., Herbinger, J., Freiesleben, T., Dandl, S., Scholbeck, C. A., Casalicchio, G., Grosse-Wentrup, M., & Bischl, B. (2022). General pitfalls of model-agnostic interpretation methods for machine learning models. In A. Holzinger, R. Goebel, R. Fong, T. Moon, K. R. Müller, & W. Samek (Eds.), xxAI - Beyond Explainable AI (Vol. 13200, p. 4). Springer. https://doi.org/10.1007/978-3-031-04083-2_4
196. Moon, K. R., van Dijk, D., Wang, Z., Gigante, S., Burkhardt, D. B., Chen, W. S., Yim, K., Elzen, A. van den, Hirn, M. J., Coifman, R. R., Ivanova, N. B., Wolf, G., & Krishnaswamy, S. (2019). Visualizing structure and transitions in high-dimensional biological data. Nat. Biotechnol., 37(12), 1482–1492. <https://doi.org/10.1038/s41587-019-0336-3>
197. Nahm, F. S. (2016). Nonparametric statistical tests for continuous data: The basic concept and the practical use. Korean J. Anesthesiol., 69(1), 8-14. https://doi.org/10.4097/kjae.2016.69.1.8
198. Nalenz, M., Rodemann, J., & Augustin, T. (2024). Learning de-biased regression trees and forests from complex samples. Mach. Learn., 113, 3379–3398. https://doi.org/10.1007/s10994-023-06439-1
199. Nazer, L. H., Zatarah, R., Waldrip, S., Ke, J. X. C., Moukheiber, M., Khanna, A. K., Hicklen, R. S., Moukheiber, L., Moukheiber, D., Ma, H., & Mathur, P. (2023). Bias in artificial intelligence algorithms and recommendations for mitigation. PLOS Digit. Health, 2(6), e0000278. https://doi.org/10.1371/journal.pdig.0000278
200. Nematzadeh, H., Enayatifar, R., Mahmud, M., & Akbari, E. (2019). Frequency based feature selection method using whale algorithm. Genomics, 111(6), 1946–1955. https://doi.org/10.1016/j.ygeno.2019.01.006
201. Newson, R. (2006). Confidence Intervals for Rank Statistics: Somers’ D and Extensions. Stata J., 6(3), 309–334. https://doi.org/10.1177/1536867X0600600302
202. Nguyen, B. H., Xue, B., & Zhang, M. (2020). A survey on swarm intelligence approaches to feature selection in data mining. Swarm Evol. Comput., 54, 100663. https://doi.org/10.1016/j.swevo.2020.100663
203. Nguyen, C. D., Strazdins, L., Nicholson, J. M., & Cooklin, A. R. (2018). Impact of missing data strategies in studies of parental employment and health: Missing items, missing waves, and missing mothers. Soc. Sci. Med., 209, 160-168. https://doi.org/10.1016/j.socscimed.2018.03.009
204. Nguyen, T. T., Huang, J. Z., & Nguyen, T. T. (2015). Unbiased feature selection in learning random forests for high-dimensional data. Sci. World J., 2015, Article 471371. https://doi.org/10.1155/2015/471371
205. Nogueira, M. S., Barreto, A. L., Furukawa, M., Rovai, E. S., Bastos, A., Bertoncello, G., Silva de Carvalho, L. F. C. (2022). FTIR spectroscopy as a point of care diagnostic tool for diabetes and periodontitis: A saliva analysis approach. Photodiagnosis and Photodynamic Therapy, 40, 103036. https://doi.org/10.1016/j.pdpdt.2022.103036
206. Nyamundanda, G., Brennan, L., & Gormley, I. C. (2010). Probabilistic principal component analysis for metabolomic data. BMC Bioinformatics, 11, Article 571. https://doi.org/10.1186/1471-2105-11-571
207. O’Brien, T. E., & Silcox, J. W. (2024). Nonlinear regression modelling: A primer with applications and caveats. Bull. Math. Biol., 86, 40. https://doi.org/10.1007/s11538-024-01274-4
208. O’Driscoll, D., & Ramirez, D. E. (2015). Response surface designs using the generalized variance inflation factors. Cogent Math., 2(1). https://doi.org/10.1080/23311835.2015.1053728
209. Okoye, K., & Hosseini, S. (2024). Correlation Tests in R: Pearson Cor, Kendall’s Tau, and Spearman’s Rho. In R Program. (pp. 205-220). Springer, Singapore. https://doi.org/10.1007/978-981-97-3385-9_12
210. Owoeye, O. R., Oluwole, A. M., Jolayemi, O. S., & Oluwalana, I. B. (2023). Linear and nonlinear regression modeling of the chemical, physical and quality variations in Cardaba banana (Musa acuminata x balbisiana – ABB) during ripening. Food Measure, 17, 12–23. https://doi.org/10.1007/s11694-022-01570-4
211. Pantazatos, S. P., Talati, A., Pavlidis, P., & Hirsch, J. (2012). Cortical functional connectivity decodes subconscious, task-irrelevant threat-related emotion processing. Neuroimage, 61(4), 1355-1363. https://doi.org/10.1016/j.neuroimage.2012.03.051
212. Parmeter, C. F., & Zhao, S. (2024). An alternative corrected ordinary least squares estimator for the stochastic frontier model. In S. C. Kumbhakar, R. C. Sickles, & H. J. Wang (Eds.), Adv. Appl. Econom. (Vol. 55, pp. 355-375). Springer, Cham. https://doi.org/10.1007/978-3-031-48385-1_15
213. Paul, D., Sinnarasan, V. S. P., Das, R., Sheikh, M. M. R., & Venkatesan, A. (2024). Machine learning approach to predict blood-secretory proteins and potential biomarkers for liver cancer using omics data. J. Proteomics, 309, 105298. https://doi.org/10.1016/j.jprot.2024.105298
214. Peralta, B., & Soto, A. (2014). Embedded local feature selection within mixture of experts. Inf. Sci., 269, 176-187. https://doi.org/10.1016/j.ins.2014.01.008
215. Peralta, M., Jannin, P., Haegelen, C., & Baxter, J. S. H. (2021). Data imputation and compression for Parkinson's disease clinical questionnaires. Artif. Intell. Med., 114, 102051. https://doi.org/10.1016/j.artmed.2021.102051
216. Perreault, S. (2024). Simultaneous computation of Kendall’s tau and its jackknife variance. Stat. Probab. Lett., 213, 110181. https://doi.org/10.1016/j.spl.2024.110181
217. Pfeifer, B., Gevaert, A., Loecher, M., & Holzinger, A. (2025). Tree smoothing: Post-hoc regularization of tree ensembles for interpretable machine learning. Inf. Sci., 690, 121564. https://doi.org/10.1016/j.ins.2024.121564
218. Pinheiro-Guedes, L., Martinho, C., & Martins, M. R. (2024). Logistic regression: Limitations in the estimation of measures of association with binary health outcomes. Acta Médica Portuguesa, 37(10), 697-705. https://doi.org/10.20344/amp.21435
219. Politi, M. T., Ferreira, J. C., & Patino, C. M. (2021). Nonparametric statistical tests: Friend or foe? Jornal Brasileiro de Pneumologia, 47(4), e20210292. https://doi.org/10.36416/1806-3756/e20210292
220. Potharlanka, J. L., & Bhat M, N. (2024). Feature importance feedback with Deep Q process in ensemble-based metaheuristic feature selection algorithms. Sci. Rep., 14(1), 2923. https://doi.org/10.1038/s41598-024-53141-w
221. Prasad, S., & Bruce, L. M. (2008). Limitations of Principal Components Analysis for Hyperspectral Target Recognition. IEEE Geoscience and Remote Sensing Letters, 5(4), 625-629. https://doi.org/10.1109/LGRS.2008.2001282
222. Qi, X., Lu, Y., Shi, Y., Qi, H., & Ren, L. (2024). A deep neural network prediction method for diabetes based on Kendall's correlation coefficient and attention mechanism. PLoS One, 19(7), e0306090. https://doi.org/10.1371/journal.pone.0306090
223. Qian, H., Wang, B., Yuan, M., Gao, S., & Song, Y. (2022). Financial distress prediction using a corrected feature selection measure and gradient boosted decision tree. Expert Syst. Appl., 190, 116202. https://doi.org/10.1016/j.eswa.2021.116202
224. Qu, W., Zhou, Z., Yuan, G., Li, S., Li, J., Chu, Q., Zhang, Q., Xie, Q., Li, Z., & Kamel, I. R. (2023). Is the radiomics-clinical combined model helpful in distinguishing between pancreatic cancer and mass-forming pancreatitis? Eur. J. Radiol., 164, 110857. https://doi.org/10.1016/j.ejrad.2023.110857
225. Racette, L., Chiou, C. Y., Hao, J., Bowd, C., Goldbaum, M. H., Zangwill, L. M., Lee, T.-W., Weinreb, R. N., & Sample, P. A. (2010). Combining functional and structural tests improves the diagnostic accuracy of relevance vector machine classifiers. Journal of Glaucoma, 19(3), 167-175.1 https://doi.org/10.1097/IJG.0b013e3181a98b85
226. Raudys, S., & Pikelis, V. (1982). Collective selection of the best version of a pattern recognition system. Pattern Recognit. Letters, 1(1), 7-13. https://doi.org/10.1016/0167-8655(82)90044-7
227. Raykov, Y. P., Boukouvalas, A., Baig, F., & Little, M. A. (2016). What to do when K-means clustering fails: A simple yet principled alternative algorithm. PLoS One, 11(9), e0162259. https://doi.org/10.1371/journal.pone.0162259
228. Rifada, M., Chamidah, N., & Ningrum, R. A. (2022). Estimation of nonparametric ordinal logistic regression model using generalized additive models (GAM) method based on local scoring algorithm. AIP Conf. Proc., 2668(1), 070013. https://doi.org/10.1063/5.0111771
229. Ringle, C. M., Sarstedt, M., Sinkovics, N., & Sinkovics, R. R. (2023). A perspective on using partial least squares structural equation modelling in data articles. Data Brief, 48, 109074. https://doi.org/10.1016/j.dib.2023.109074
230. Ros, F., Riad, R., & Guillaume, S. (2023). PDBI: A partitioning Davies-Bouldin index for clustering evaluation. Neurocomputing, 528, 178-199. https://doi.org/10.1016/j.neucom.2023.01.043
231. Roussos, G., Ruiz Herrero, T., Hill, D. L., Dowling, A. V., Müller, M. L. T. M., Evers, L. J. W., Burton, J., Derungs, A., Fisher, K., Kilambi, K. P., Mehrotra, N., Bhatnagar, R., Sardar, S., Stephenson, D., Adams, J. L., Dorsey, E. R., & Cosman, J. (2022). Identifying and characterising sources of variability in digital outcome measures in Parkinson's disease. NPJ Digit. Med., 5(1), 93. Published 2022 Jul 15. https://doi.org/10.1038/s41746-022-00643-4
232. Saccenti, E., Hendriks, M. H. W. B., & Smilde, A. K. (2020). Corruption of the Pearson correlation coefficient by measurement error and its estimation, bias, and correction under 1 different error models. Sci. Rep., 10, 438. https://doi.org/10.1038/s41598-019-57247-4
233. Sahran, S., Albashish, D., Abdullah, A., Shukor, N. A., & Pauzi, S. H. (2018). Absolute cosine-based SVM-RFE feature selection method for prostate histopathological grading. Artif. Intell. Med., 87, 78-90. https://doi.org/10.1016/j.artmed.2018.04.002
234. Sahu, P., Kang, J., Erdemci-Tandogan, G., & Manning, M. L. (2020). Linear and nonlinear mechanical responses can be quite different in models for biological tissues. Soft Matter, 16(7), 1850-1856. https://doi.org/10.1039/c9sm01068h
235. Salles, T., Rocha, L., & Gonçalves, M. (2021). A bias-variance analysis of state-of-the-art random forest text classifiers. Adv. Data Anal. Classif., 15, 379-405. https://doi.org/10.1007/s11634-020-00409-4
236. Salmerón-Gómez, R., García-García, C. B., & García-Pérez, J. (2025). A redefined variance inflation factor: Overcoming the limitations of the variance inflation factor. Computational Economics, 65, 337–363. https://doi.org/10.1007/s10614-024-10575-8
237. Samuel, D., Boboc, B., Bernuau, J., Bismuth, H., & Benhamou, J. P. (1988). Liver Transplantation for Protoporphyria: Evidence for the Predominant Role of the Erythropoietic Tissue in Protoporphyrin Overproduction. Gastroenterology, 95(3), 816-819. https://doi.org/10.1016/S0016-5085(88)80033-7
238. Sanjalawe, Y., & Althobaiti, T. (2023). DDoS Attack Detection in Cloud Computing Based on Ensemble Feature Selection and Deep Learning. Computers, Materials and Continua, 75(2), 3571-3588. https://doi.org/10.32604/cmc.2023.037386
239. Sarstedt, M., Ringle, C. M., Cheah, J.-H., Ting, H., Moisescu, O. I., & Radomir, L. (2019). Structural model robustness checks in PLS-SEM. Tourism Economics, 26(4), 531–554. https://doi.org/10.1177/1354816618823921 (Original work published 2020)
240. Schober, P., & Vetter, T. R. (2020). Nonparametric statistical methods in medical research. Anesthesia & Analgesia, 131(6), 1862-1863. https://doi.org/10.1213/ANE.0000000000005101
241. Schwarzer, A., Talbot, S. R., Selich, A., Morgan, M., Schott, J. W., Dittrich-Breiholz, O., Bastone, A. L., Weigel, B., Ha, T. C., Dziadek, V., Gijsbers, R., Thrasher, A. J., Staal, F. J. T., Gaspar, H. B., Modlich, U., Schambach, A., & Rothe, M. (2021). Predicting genotoxicity of viral vectors for stem cell gene therapy using gene expression-based machine learning. Mol. Ther., 29(12), 3383-3397. https://doi.org/10.1016/j.ymthe.2021.06.017
242. Searcy, R. T., Phaneuf, J. R., & Boehm, A. B. (2023). High-frequency fecal indicator bacteria (FIB) observations to assess water quality drivers at an enclosed beach. PLoS One, 18(6), e0286029. Published 2023 Jun 2. https://doi.org/10.1371/journal.pone.0286029
243. Sefidian, A. M., & Daneshpour, N. (2019). Missing value imputation using a novel grey based fuzzy c-means, mutual information based feature selection, and regression model. Expert Syst. Appl., 115, 68-94. https://doi.org/10.1016/j.eswa.2018.07.057
244. Sefidian, A. M., & Daneshpour, N. (2020). Estimating missing data using novel correlation maximization based methods. Appl. Soft Comput., 91, 106249. https://doi.org/10.1016/j.asoc.2020.106249
245. Semwal, R., & Varadwaj, P. K. (2020). HumDLoc: Human Protein Subcellular Localization Prediction Using Deep Neural Network. Current Genomics, 21(7), 546-557. https://doi.org/10.2174/1389202921999200528160534
246. Shahapure, K. R., & Nicholas, C. (2020). Cluster quality analysis using Silhouette Score. In Proceedings of the 2020 IEEE 7th International Conference on Data Science and Advanced Analytics (DSAA) (pp. 747-748). Sydney, NSW, Australia. https://doi.org/10.1109/DSAA49011.2020.00096
247. Shao, Z., Moon, Y. B., Yang, Y. C., & Kumral, M. (2023). Underground haulage network design using HDBSCAN and RRT algorithms built on Dubins path. Mining, Metallurgy & Exploration, 40, 773–786. https://doi.org/10.1007/s42461-023-00777-3
248. Shen, H., Bhamidi, S., & Liu, Y. (2024). Statistical significance of clustering with multidimensional scaling. Journal of Computational and Graphical Statistics, 33(1), 219-230. https://doi.org/10.1080/10618600.2023.2219708
249. Shen, Y., Lu, Q., Zhang, T., Yan, H., Mansouri, N., Osipowicz, K., Tanglay, O., Young, I., Doyen, S., Lu, X., Zhang, X., Sughrue, M. E., & Wang, T. (2022). Use of machine learning to identify functional connectivity changes in a clinical cohort of patients at risk for dementia. Frontiers in Aging Neuroscience, 14, 962319. Published 2022 Sep 1. https://doi.org/10.3389/fnagi.2022.962319
250. Shi, L., Westerhuis, J. A., Rosén, J., Landberg, R., & Brunius, C. (2019). Variable selection and validation in multivariate modelling. Bioinformatics, 35(6), 972-980. https://doi.org/10.1093/bioinformatics/bty710
251. Shi, Y., Golestanian, R., & Vilfan, A. (2024). Mutual information as a measure of mixing efficiency in viscous fluids. Physical Review Research, 6(2), L022050. https://doi.org/10.1103/PhysRevResearch.6.L022050
252. Shin, H., & Markey, M. K. (2006). A machine learning perspective on the development of clinical decision support systems utilizing mass spectra of blood samples. Journal of Biomedical Informatics, 39(2), 227-248. https://doi.org/10.1016/j.jbi.2005.04.002
253. Shutaywi, M., & Kachouie, N. N. (2021). Silhouette Analysis for Performance Evaluation in Machine Learning with Applications to Clustering. Entropy, 23(6), 759. https://doi.org/10.3390/e23060759
254. Smart, S. E., Agbedjro, D., Pardiñas, A. F., Ajnakina, O., Alameda, L., Andreassen, O. A., Barnes, T. R. E., Berardi, D., Camporesi, S., Cleusix, M., Conus, P., Crespo-Facorro, B., D’Andrea, G., Demjaha, A., Di Forti, M., Do, K., Doody, G., Eap, C. B., Ferchiou, A., & Guidi, L. (2022). Clinical predictors of antipsychotic treatment resistance: Development and internal validation of a prognostic prediction model by the STRATA-G consortium. Schizophrenia Research, 250, 1-9. https://doi.org/10.1016/j.schres.2022.09.009
255. Smith, H. L., Biggs, P. J., French, N. P., Smith, A. N. H., & Marshall, J. C. (2024). Lost in the Forest: Encoding categorical variables and the absent levels problem. Data Min. Knowl. Discov., 38, 1889-1908. https://doi.org/10.1007/s10618-024-01019-w
256. Song, Q., Merajver, S. D., & Li, J. Z. (2015). Cancer classification in the genomic era: five contemporary problems. Human Genomics, 9, 27. Published 2015 Oct 19. https://doi.org/10.1186/s40246-015-0049-8
257. Song, X. F., Zhang, Y., Gong, D. W., & Sun, X. Y. (2021). Feature selection using bare-bones particle swarm optimization with mutual information. Pattern Recognit., 112, 107804. https://doi.org/10.1016/j.patcog.2020.107804
258. Stamate, C., Saez Pons, J., Weston, D., & Roussos, G. (2021). PDKit: A data science toolkit for the digital assessment of Parkinson's Disease. PLoS Computational Biology, 17(3), e1008833. Published 2021 Mar 12. https://doi.org/10.1371/journal.pcbi.1008833
259. Steiger, J. H. (2007). Understanding the limitations of global fit assessment in structural equation modeling. Personality and Individual Differences, 42(5), 893-898. https://doi.org/10.1016/j.paid.2006.09.017
260. Steiner, P. M., & Kim, Y. (2016). The mechanics of omitted variable bias: Bias amplification and cancellation of offsetting biases. Journal of Causal Inference, 4(2), 20160009. https://doi.org/10.1515/jci-2016-0009
261. Stiglic, G., Povalej Brzan, P., Fijacko, N., Wang, F., Delibasic, B., Kalousis, A., & Obradovic, Z. (2015). Comprehensible Predictive Modeling Using Regularized Logistic Regression and Comorbidity Based Features. PLoS One, 10(12), e0144439. Published 2015 Dec 8. https://doi.org/10.1371/journal.pone.0144439
262. Stiglic, G., Rodriguez, J. J., & Kokol, P. (2010). Finding optimal classifiers for small feature sets in genomics and proteomics. Neurocomputing, 73(13-15), 2346-2352. https://doi.org/10.1016/j.neucom.2010.02.024
263. Stojanova, D., Ceci, M., Appice, A., Malerba, D., & Džeroski, S. (2013). Dealing with spatial autocorrelation when learning predictive clustering trees. Ecol. Inform., 13, 22-39. https://doi.org/10.1016/j.ecoinf.2012.10.006
264. Strobl, C., Boulesteix, A. L., Zeileis, A., & Hothorn, T. (2007). Bias in random forest variable importance measures: illustrations, sources and a solution. BMC Bioinformatics, 8, 25. https://doi.org/10.1186/1471-2105-8-25
265. Suárez-Marcote, S., Morán-Fernández, L., & Bolón-Canedo, V. (2024). Towards federated feature selection: Logarithmic division for resource-conscious methods. Neurocomputing, 596, 128099. https://doi.org/10.1016/j.neucom.2024.128099
266. Sun, J., & Li, H. (2008). Data mining method for listed companies’ financial distress prediction. Knowl. Based Syst., 21(1), 1-5. https://doi.org/10.1016/j.knosys.2006.11.003
267. Tagiling, N., Lee, Y. Y., Mohd Rohani, M. F., Udin, M. Y., Abdul Aziz, A., Muhamad, S. N., Musarudin, M., Abdul Razab, M. K. A., Zainol, N. A. S., Tan, P. O., & Mat Nawi, N. (2025). Gastric accommodation testing using hybrid nuclear imaging volumetry and combined high-resolution manometry-nutrient drink test: A pilot study in healthy individuals. Neurogastroenterology & Motility. Advance online publication. https://doi.org/10.1111/nmo.15006
268. Takefuji, Y. (2024). Mitigating biases in feature selection and importance assessments in predictive models using LASSO regression. Oral Oncology, 159, 107090. https://doi.org/10.1016/j.oraloncology.2024.107090
269. Tang, C. Y., Gao, C., Prasai, K., Li, T., Dash, S., McElroy, J. A., Hang, J., & Wan, X.-F. (2024). Prediction models for COVID-19 disease outcomes. Emerging Microbes & Infections, 13(1), 2361791. https://doi.org/10.1080/22221751.2024.2361791
270. Tang, L. J., Li, X. K., Huang, Y., Zhang, X. Z., & Li, B. Q. (2024). Accurate and visualiable discrimination of Chenpi age using 2D-CNN and Grad-CAM++ based on infrared spectral images. Food Chemistry: X, 23, 101759. https://doi.org/10.1016/j.fochx.2024.101759
271. Tang, W., Zhang, Q., Chen, Y., Liu, X., Wang, H., & Huang, W. (2024). An intelligent airflow perception model for metal mines based on CNN-LSTM architecture. Process Safety and Environmental Protection, 187, 1234-1247. https://doi.org/10.1016/j.psep.2024.05.044
272. Tarabichi, M., Saiselet, M., Trésallet, C., Hoang, C., Larsimont, D., Andry, G., Maenhaut, C., & Detours, V. (2015). Revisiting the transcriptional analysis of primary tumours and associated nodal metastases with enhanced biological and statistical controls: application to thyroid cancer. British Journal of Cancer, 112(10), 1665-1674. https://doi.org/10.1038/bjc.2014.665
273. Taylor, J. A., Larsen, K. M., Dzafic, I., & Garrido, M. I. (2021). Predicting subclinical psychotic-like experiences on a continuum using machine learning. Neuroimage, 241, 118329. https://doi.org/10.1016/j.neuroimage.2021.118329
274. Tekchandani, H., Verma, S., & Londhe, N. (2020). Performance improvement of mediastinal lymph node severity detection using GAN and Inception network. Comput. Methods Programs Biomed., 194, 105478. https://doi.org/10.1016/j.cmpb.2020.105478
275. Thakur, D., & Biswas, S. (2024). Permutation importance based modified guided regularized random forest in human activity recognition with smartphone. Eng. Appl. Artif. Intell., 129, 107681. https://doi.org/10.1016/j.engappai.2023.107681
276. Thanjavur, K., Hristopulos, D. T., Babul, A., Yi, K. M., & Virji-Babul, N. (2021). Deep Learning Recurrent Neural Network for Concussion Classification in Adolescents Using Raw Electroencephalography Signals: Toward a Minimal Number of Sensors. Frontiers in Human Neuroscience, 15, 734501. Published 2021 Nov 24. https://doi.org/10.3389/fnhum.2021.734501
277. Thomson, T. M., Lescarbeau, R. M., Drubin, D. A., Laifenfeld, D., de Graaf, D., Fryburg, D. A., Littman, B., Deehan, R., & Van Hooser, A. (2015). Blood-based identification of non-responders to anti-TNF therapy in rheumatoid arthritis. BMC Medical Genomics, 8, 26. Published 2015 Jun 3. http://doi.org/10.1186/s12920-015-0100-6
278. Timmons, A. C., Duong, J. B., Fiallo, N. S., Lee, T., Vo, H. P. Q., Ahle, M. W., Comer, J. S., Brewer, L. C., Frazier, S. L., & Chaspari, T. (2023). A Call to Action on Assessing and Mitigating Bias in Artificial Intelligence Applications for Mental Health. Perspectives on Psychological Science, 18(5), 1062-1096. https://doi.org/10.1177/17456916221134490
279. Tomalin, L. E., Kim, J., Correa da Rosa, J., Lee, J., Fitz, L. J., Berstein, G., Valdez, H., Wolk, R., Krueger, J. G., & Suárez-Fariñas, M. (2020). Early Quantification of Systemic Inflammatory Proteins Predicts Long-Term Treatment Response to Tofacitinib and Etanercept. Journal of Investigative Dermatology, 140(5), 1026-1034. https://doi.org/10.1016/j.jid.2019.09.023
280. Tomarken, A. J., & Waller, N. G. (2005). Structural equation modeling: strengths, limitations, and misconceptions. Annual Review of Clinical Psychology, 1, 31–65. https://doi.org/10.1146/annurev.clinpsy.1.102803.144239
281. Torres Moral, T., Sanchez-Niubo, A., Monistrol-Mula, A., Gerardi, C., Banzi, R., Garcia, P., Demotes-Mainard, J., Haro, J. M., & the PERMIT Group. (2022). Methods for Stratification and Validation Cohorts: A Scoping Review. Journal of Personalized Medicine, 12(5), 688. Published 2022 Apr 26. https://doi.org/10.3390/jpm12050688
282. Tracy, S., Yuan, G. C., & Dries, R. (2019). RESCUE: imputing dropout events in single-cell RNA-sequencing data. BMC Bioinformatics, 20(1), 388. Published 2019 Jul 12. https://doi.org/10.1186/s12859-019-2977-0
283. Tran, H. T. T., Van den Bergh, R., Vu, T. N., Laukens, K., Worodria, W., Loembé, M. M., Colebunders, R., Kestens, L., De Baetselier, P., & Raes, G. (2014). The role of monocytes in the development of Tuberculosis-associated Immune Reconstitution Inflammatory Syndrome. Immunobiology, 219(1), 37-44. https://doi.org/10.1016/j.imbio.2013.07.004
284. Tserkis, S., Assad, S. M., Lam, P. K., & Narang, P. (2025). Quantifying total correlations in quantum systems through the Pearson correlation coefficient. Physics Letters A, 543, 130432. https://doi.org/10.1016/j.physleta.2025.130432
285. Ugirumurera, J., Bensen, E. A., Severino, J., & Sanyal, J. (2024). Addressing bias in bagging and boosting regression models. Sci. Rep., 14(1), 18452. https://doi.org/10.1038/s41598-024-68907-5
286. Ugrinowitsch, C., Fellingham, G. W., & Ricard, M. D. (2004). Limitations of ordinary least squares models in analyzing repeated measures data. Med. Sci. Sports Exerc., 36(12), 2144–2148. https://doi.org/10.1249/01.mss.0000147580.40591.75
287. Umeki, N., Kabashima, Y., & Sako, Y. (2025). Evaluation of information flows in the RAS-MAPK system using transfer entropy measurements. eLife, 14, e104432. https://doi.org/10.7554/eLife.104432
288. Ünal, B. (2022). Causality analysis for COVID-19 among countries using effective transfer entropy. Entropy, 24(8), 1115. https://doi.org/10.3390/e24081115
289. Vable, A. M., Kiang, M. V., Glymour, M. M., Rigdon, J., Drabo, E. F., & Basu, S. (2019). Performance of Matching Methods as Compared With Unmatched Ordinary Least Squares Regression Under Constant Effects. American Journal of Epidemiology, 188(7), 1345–1354. https://doi.org/10.1093/aje/kwz093
290. van Koppen, A., Verschuren, L., van den Hoek, A. M., Verheij, J., Morrison, M. C., Li, K., Nagabukuro, H., Costessi, A., Caspers, M. P. M., van den Broek, T 1 . J., Sagartz, J., Kluft, C., Beysen, C., Emson, C., van Gool, A. J., Goldschmeding, R., Stoop, R., Bobeldijk-Pastorova, I., Turner, S. M., Hanauer, G., & Hanemaaijer, R. (12018). Uncovering a predictive molecular signature for the onset of NASH-related fibrosis in a translational NASH mouse model. Cellular and Molecular Gastroenterology and Hepatolog2y, 5(1), 83-98.e10. https://doi.org/10.1016/j.jcmgh.2017.10.001
291. van Maanen, L., Katsimpokis, D., & van Campen, A. D. (2019). Fast and slow errors: Logistic regression to identify patterns in accuracy–response time relationships. Behav. Res. Methods, 51, 2378–2389. https://doi.org/10.3758/s13428-018-1110-z
292. Varley, T. F., Pope, M., & Sporns, O. (2023). Partial entropy decomposition reveals higher-order information structures in human brain activity. Proc. Natl. Acad. Sci. USA, 120(30), e2300888120. https://doi.org/10.1073/pnas.2300888120
293. Vos, S., Hebeda, K., Milota, M., Sand, M., Drogt, J., Grünberg, K., Jongsma, K. (2024). Making pathologists ready for the new AI era: changes in required competencies. Modern Pathology, 100657. https://doi.org/10.1016/j.modpat.2024.100657
294. Waernbaum, I., & Pazzagli, L. (2023). Model misspecification and bias for inverse probability weighting estimators of average causal effects. Biometrical Journal. Biometrische Zeitschrift, 65(2), e2100118. https://doi.org/10.1002/bimj.202100118
295. Wallace, M. L., Mentch, L., Wheeler, B. J., Lyons, M., & Reichmann, W. M. (2023). Use and misuse of random forest variable importance metrics in medicine: demonstrations through incident stroke prediction. BMC Med. Res. Methodol., 23(1), 144. https://doi.org/10.1186/s12874-023-01965-x
296. Wang, H., Lo, S. H., Zheng, T., & Hu, I. (2012). Interaction-based feature selection and classification for high-dimensional biological data. Bioinformatics, 28(21), 2834-2842. https://doi.org/10.1093/bioinformatics/bts531
297. Wang, T., Tang, W., Lin, Y., & Su, W. (2023). Semi-supervised inference for nonparametric logistic regression. Stat. Med., 42(15), 2573–2589. https://doi.org/10.1002/sim.9737
298. Wei, W., Li, Y., & Huang, T. (2023). Using Machine Learning Methods to Study Colorectal Cancer Tumor Micro-Environment and Its Biomarkers. Int. J. Mol. Sci., 24(13), 11133. Published 2023 Jul 6. https://doi.org/10.3390/ijms241311133
299. Weintraub, M. J., Posta, F., Arevian, A. C., & Miklowitz, D. J. (2021). Using machine learning analyses of speech to classify levels of expressed emotion in parents of youth with mood disorders. J. Psychiatr. Res., 136, 39-46. https://doi.org/10.1016/j.jpsychires.2021.01.019
300. Wesolowski, C. A., Puetter, R. C., Babyn, P. S., & Ling, L. (2010). Limitations of ordinary least squares fitting of gamma variate functions to plasma-clearance curves. J Nucl Med, 51(Supplement 2), No.1674. https://doi.org/10.1007/s10928-010-9167-z
301. Wongoutong, C. (2024). The impact of neglecting feature scaling in k-means clustering. PLoS One, 19(12), e0310839. https://doi.org/10.1371/journal.pone.0310839
302. Wood, D., Papamarkou, T., Benatan, M., & Allmendinger, R. (2024). Model-agnostic variable importance for predictive uncertainty: An entropy-based approach. Data Min. Knowl. Discov., 38, 4184-4216. https://doi.org/10.1007/s10618-024-01070-7
303. Work, J. W., Ferguson, J. G., & Diamond, G. A. (1989). Limitations of a conventional logistic regression model based on left ventricular ejection fraction in predicting coronary events after myocardial infarction. American Journal of Cardiology, 64(12), 702-707. https://doi.org/10.1016/0002-9149(89)90751-0
304. Wüthrich, K., & Zhu, Y. (2023). Omitted variable bias of Lasso-based inference methods: A finite sample analysis. Review of Economics and Statistics, 105(4), 982–997. https://doi.org/10.1162/rest_a_01128
305. Xie, Y. R., Castro, D. C., Bell, S. E., Rubakhin, S. S., & Sweedler, J. V. (2020). Single-Cell Classification Using Mass Spectrometry through Interpretable Mach. Learn.. Analytical Chemistry, 92(13), 9338-9347. https://doi.org/10.1021/acs.analchem.0c01660
306. Xu, F., Kong, F., Peng, H., Dong, S., Gao, W., & Zhang, G. (2021). Combing machine learning and elemental profiling for geographical authentication of Chinese Geographical Indication (GI) rice. NPJ Science of Food, 5(1), 18. Published 2021 Jul 8. https://doi.org/10.1038/s41538-021-00100-8
307. Xu, H., Newlin, N. R., Kim, M. E., Gao, C., Kanakaraj, P., Krishnan, A. R., Remedios, L. W., Khairi, N. M., Pechman, K., Archer, D., Hohman, T. J., Jefferson, A. L., Isgum, I., Huo, Y., Moyer, D., Schilling, K. G., & Landman, B. A. (2024). Evaluation of mean shift, ComBat, and CycleGAN for harmonizing brain connectivity matrices across sites. In Proceedings of SPIE (Vol. 12926, Article 129261X). https://doi.org/10.1117/12.3005563
308. Xu, K., Cheng, Q., & He, D. (2025). On summed nonparametric dependence measures in high dimensions, fixed or large samples. Comput. Stat. & Data Analysis, 205, 108109. https://doi.org/10.1016/j.csda.2024.108109
309. Xu, X., Liang, T., Zhu, J., Zheng, D., & Sun, T. (2019). Review of classical dimensionality reduction and sample selection methods for large-scale data processing. Neurocomputing, 328, 5-15. https://doi.org/10.1016/j.neucom.2018.02.100
310. Yang, G., He, J., Lan, X., Li, T., & Fang, W. (2024). A fast dual-module hybrid high-dimensional feature selection algorithm. Inf. Sci., 681, 121185. https://doi.org/10.1016/j.ins.2024.121185
311. Yang, G., Li, W., Xie, W., Wang, L., & Yu, K. (2024). An improved binary particle swarm optimization algorithm for clinical cancer biomarker identification in microarray data. Comput. Methods Programs Biomed., 244, 107987. https://doi.org/10.1016/j.cmpb.2023.107987
312. Yang, Y., & Webb, G. I. (2009). Discretization for naive-Bayes learning: Managing discretization bias and variance. Mach. Learn., 74, 39–74. https://doi.org/10.1007/s10994-008-5083-5
313. Yao, Y., & Ochoa, A. (2023). Limitations of principal components in quantitative genetic association models for human studies. eLife, 12, Article e79238. https://doi.org/10.7554/eLife.79238
314. Yip, S. S., & Aerts, H. J. (2016). Applications and limitations of radiomics. Phys. Med. Biol., 61(13), R150-R166. https://doi.org/10.1088/0031-9155/61/13/R150
315. Yu, B., Cho, J., Kang, B. H., Kim, K., Kim, D. H., Chang, S. W., Jung, P. Y., Heo, Y., & Kang, W. S. (2024). Nomogram for predicting in-hospital mortality in trauma patients undergoing resuscitative endovascular balloon occlusion of the aorta: a retrospective multicenter study. Sci. Rep., 14(1), 9164. https://doi.org/10.1038/s41598-024-59861-3
316. Yu, F., Wei, C., Deng, P., Peng, T., & Hu, X. (2021). Deep exploration of random forest model boosts the interpretability of machine learning studies of complicated immune responses and lung burden of nanoparticles. Science Advances, 7(22), eabf4130. Published 2021 May 26. https://doi.org/10.1126/sciadv.abf4130
317. Yu, H., & Hutson, A. D. (2024). A robust Spearman correlation coefficient permutation test. Communications in Statistics: Theory and Methods, 53(6), 2141-2153. https://doi.org/10.1080/03610926.2022.2121144
318. Yu, K., Sun, S., Liang, J., Chen, K., Qu, B., Yue, C., & Wang, L. (2023). A bidirectional dynamic grouping multi-objective evolutionary algorithm for feature selection on high-dimensional classification. Inf. Sci., 648, 119619. https://doi.org/10.1016/j.ins.2023.119619
319. Zajac, G., & Ignatiev, A. (1979). High temperature optical and structural degradation of black chrome coatings. Solar Energy Materials, 2(2), 239-247. https://doi.org/10.1016/0165-1633(79)90021-2
320. Zarei, M., Najarchi, M., & Mastouri, R. (2021). Bias correction of global ensemble precipitation forecasts by Random Forest method. Earth Science Informatics, 14, 677-689. https://doi.org/10.1007/s12145-021-00577-7
321. Zemariam, A. B., Yimer, A., Abebe, G. K., Wondie, W. T., Abate, B. B., Alamaw, A. W., Yilak, G., Melaku, T. M., & Ngusie, H. S. (2024). Employing supervised machine learning algorithms for classification and prediction of anemia among youth girls in Ethiopia. Sci. Rep., 14(1), 9080. Published 2024 Apr 20. https://doi.org/10.1038/s41598-024-60027-4
322. Zhang, F., Xu, Y., & Yuan, D. (2024). Detecting financial contagion using a new nonparametric measure of asymmetric comovements. Int. Rev. Econ. Finance, 89, 284-296. https://doi.org/10.1016/j.iref.2023.07.067
323. Zhang, H., Tan, S., Chen, W., Kligerman, S., Kim, G., D'Souza, W. D., Suntharalingam, M., & Lu, W. (2014). Modeling pathologic response of esophageal cancer to chemoradiation therapy using spatial-temporal 18F-FDG PET features, clinical parameters, and demographics. International Journal of Radiation Oncology•Biology•Physics, 88(1), 195-203. https://doi.org/10.1016/j.ijrobp.2013.09.037
324. Zhang, R., Zhang, R., Luan, T., Liu, B., Zhang, Y., Xu, Y., Sun, X., & Xing, L. (2021). A Radiomics Nomogram for Preoperative Prediction of Clinical Occult Lymph Node Metastasis in cT1-2N0M0 Solid Lung Adenocarcinoma. Cancer Manag. Res., 13, 8157-8167. Published 2021 Oct 28. https://doi.org/10.2147/CMAR.S330824
325. Zhang, W., Yue, Z., Ye, J., Xu, H., Wang, Y., Zhang, X., & Xi, L. (2022). Modulation format identification using the Calinski–Harabasz index. Appl. Opt., 61(3), 851-857. https://doi.org/10.1364/ao.448043
326. Zhang, Y., Nie, B., Du, J., Chen, J., Du, Y., Jin, H., Zheng, X., Chen, X., & Miao, Z. (2023). Feature selection based on neighborhood rough sets and Gini index. PeerJ Computer Science, 9, e1711. Published 2023 Dec 12. https://doi.org/10.7717/peerj-cs.1711
327. Zhang, Y., Song, X., & Gong, D. (2017). A return-cost-based binary firefly algorithm for feature selection. Inf. Sci., 418-419, 561-574. https://doi.org/10.1016/j.ins.2017.08.047
328. Zhao, F., Lu, Y., Li, X., Wang, L., Song, Y., Fan, D., Zhang, C., & Chen, X. (2022). Multiple imputation method of missing credit risk assessment data based on generative adversarial networks. Appl. Soft Comput., 126, 109273. https://doi.org/10.1016/j.asoc.2022.109273
329. Zhou, L., Lu, D., & Fujita, H. (2015). The performance of corporate financial distress prediction models with features selection guided by domain knowledge and data mining approaches. Knowl. Based Syst., 85, 52-61. https://doi.org/10.1016/j.knosys.2015.04.017
330. Zhou, Y., Xu, K., Zhu, L., & Li, R. (2024). Rank-based indices for testing independence between two high-dimensional vectors. Ann. Stat., 52(1), 184-206. https://doi.org/10.1214/23-aos2339
331. Zuur, A. F., Ieno, E. N., Walker, N. J., Saveliev, A. A., & Smith, G. M. (2009). Limitations of linear regression applied on ecological data. In Mixed Effects Models Extens. Ecol. R. Stat. Biol. Health (pp. 43-67). Springer, New York, NY. https://doi.org/10.1007/978-0-387-87458-6_2
